# Within-quadrant position and orientation specificity after extensive orientation discrimination learning is related to performance gains during late learning

**DOI:** 10.1371/journal.pone.0201520

**Published:** 2018-09-10

**Authors:** Gesa Lange, Eric Lowet, Mark J. Roberts, Peter De Weerd

**Affiliations:** 1 Department of Cognitive Neuroscience, Faculty of Psychology and Neuroscience, Maastricht University, Maastricht, The Netherlands; 2 Maastricht Centre for Systems Biology (MaCSBio), Maastricht University, Maastricht, The Netherlands; University of Verona, ITALY

## Abstract

The last decade has seen the emergence of new views about the mechanisms underlying specificity (or, conversely, generalization) of visual skill learning. Here, we trained participants at orientation discrimination paradigm at a peripheral position to induce position and orientation specificity and to test its underlying mechanisms. Specifically, we aimed to test whether the within-quadrant spatial gradient of generalization is determined by cortical magnification, which would show that retinotopic plasticity contributes to learning and specificity. Additionally, we aimed to test whether late parts of the learning relate differently to specificity compared to early parts. This is relevant in the context of double training papers, which suggest that rule-based mechanisms of specificity in fast, early learning also would apply to late, slower learning. Our data showed partial but significant position and orientation specificity within quadrants. Interestingly, specificity was greatest for those participants who had continued to show threshold decreases during the last five sessions of training (late, asymptotic learning). Performance gains during early learning were less related to specificity. A trend for skill to spread over larger distances towards periphery than towards central vision suggested contributions to transfer of early visual areas showing cortical magnification of central vision. Control experiments however did not support this hypothesis. In summary, our study demonstrates significant specificity after extensive perceptual learning, and indicates that asymptotic learning recruits specific mechanisms that promote specificity, and that may not be recruited yet in early parts of the learning. The contributions of different mechanisms to early and late learning suggests that following these different learning periods, generalization relies on different principles and is subjected to different limits.

## 1 Introduction

A long tradition of research has emphasized the characteristic time course of perceptual leaning as well as its specificity (or the lack of generalization/transfer). Its time course includes a few sessions of fast progress during early learning, followed by slower learning and ultimately a late period in which daily performance gains are small and progressively become even smaller [1, 2], also referred to as asymptotic learning [3]. Specificity of training-induced performance enhancement has been reported for the trained eye [4], retinotopic position [4–8], direction [9, 10], orientation [4–6, 11–14], spatial frequency [11], stimulus size [6, 13], and the requirements of the task [7, 13]. However, in the last decade, specificity as a core feature of perceptual learning has been challenged by a series of studies that demonstrated transfer of visual skills for the above-mentioned low-level stimulus characteristics [15–21].

Along with these apparently conflicting data on specificity, it has become increasingly recognized that many mechanisms can contribute to training-induced performance gains during perceptual learning [22, 23]. Sensory signals can be enhanced by the reduction of internal sensory or external (stimulus) noise [24], by the reduction of correlated internal noise during attentive task performance [25], or by a strengthening of the signal for example by a gain change [26] or a sharpening of tuning [12]. It furthermore has been debated to what extent noise reduction and signal enhancement is accomplished by plastic changes in low-level cortical areas [27], or by a reweighing and selection of the most informative inputs by higher-level read-out mechanisms [28, 29].

According to the lowest-level hypothesis [2], prolonged training will lead to plasticity at the lowest levels in the visual system where the trained stimulus dimension is best represented. When a skill involves discriminating an elementary feature of a stimulus, plastic changes involving neurons at low levels of the visual system, with small receptive fields and strong stimulus-tuning to the relevant feature are thought to lead to specificity of the trained visual skill. Neuronal recording studies [30] as well as fMRI studies [31, 32] support training-induced low-level visual plasticity. Studies inspired by the lowest-level hypothesis suggest that the plastic changes in low-level areas that contribute to specificity depend on continued performance gains during late, asymptotic learning [2, 12]. By contrast, during early visual learning, subcortical centers such as the basal ganglia [33] as well as high-level areas including temporal, fronto-parietal and cingulate regions [34–37] are involved and contribute to the processing of task context, feedback, and establishment of visuo-motor associations. Compared to low-level visual areas, high-level areas lack (precise) retinotopy and have neurons with large receptive fields [38–40]. For the basal ganglia and its subdivisions, there is evidence for topographic biases but no evidence for precise retinotopy [41–43]. Therefore, the early learning processes driven by high level cortical areas, and subcortical centers such as the basal ganglia, are not expected to be very position and stimulus specific.

According to read-out theories, training-induced performance gains do not require plastic changes in low-levels of the visual system, but instead are based on an increased efficiency of readout, e.g., by improving the selection of and access to the most informative neurons [28, 44]. The increased precision of spatial and featural selection may be aided by cooperation among both prefrontal and parietal areas including LIP and FEF [45–47], as well as the superior colliculus [48, 49]. According to a specific instantiation of readout, the reverse hierarchy theory [29, 50], easier tasks depend on read-out from relatively higher levels of the visual hierarchy, while more difficult tasks requiring finer analysis depend on read-out from neurons at lower levels, where neurons show smaller receptive fields and show precise stimulus tuning. In line with this, easy tasks (e.g., discriminating lines with a large orientation difference) have been shown to generalize, while more difficult tasks lead to more specificity for the same amount of training [50–52]. Similarly, specificity has been shown to depend on the precision of the task with which transfer is tested (transfer task) after initial training on another task [18]. Notably, because in reverse hierarchy theories, learning relates to reweighing the connections that are most relevant at increasingly lower levels of the visual hierarchy, they predict–just like the lowest-level hypothesis–that specificity will emerge during late, asymptotic learning, when the task is hard and the lowest levels of the visual system are read out. It is also possible that access to early visual areas is established from the beginning [53] but that the selection of informative neurons and reweighing of their inputs takes time and is only achieved in late learning.

The contributions to specificity from difficulty and effortful attention during skill acquisition have been confirmed by a variety of observations. It has been shown that the difficulty of the training task (i.e., the sensory challenge), but not the difficulty of the transfer task determines whether there is generalization [54]. Furthermore, it has been suggested that the number of training trials determines the degree of specificity [15, 17, 55]. This is in line with a recent report from Hung and Seitz [56], who showed that prolonged training at threshold leads to retinotopic specificity. The importance of repeated presentations of the training stimuli is also in line with a contribution of adaptation to learning and specificity as proposed by Harris, Gliksberg and Sagi [57], who showed that when adaptation was prevented, position specificity was abolished. Attentional mechanisms also contribute strongly to perceptual learning [1, 4, 13, 58].

Hence, irrespective of their theoretical viewpoints, there are many studies indicating that perceptual skills can become specific to stimulus features and position after sufficiently long (asymptotic) learning in a task that pushes the limits of the system. This concept has been challenged however in orientation discrimination experiments in which training at a given position largely generalized to another position if that position was pre-exposed to the stimulus in the context of an irrelevant task [20, 21, 59]. Moreover, an additional study [60] also showed that a brief pre-test was sufficient to enable substantial transfer from foveal to peripheral positions in an orientation discrimination task using Gabor stimuli and unidimensional noise fields as used by Schoups et al. [8]. These new findings of substantial generalization of orientation discrimination across trained and exposed positions were interpreted as a challenge to the view that plasticity in retinotopic areas could contribute to position specificity, as had been supported by prior studies [8, 12]. Xiao et al. [21] suggested that perceptual learning “involves higher nonretinotopic brain areas that enable location transfer” (p.1922), which would suggest a complete transfer of training-induced read-out enhancement across the visual field. As such, this explanation is different from both classical read-out theories in which high-level areas interact with low-level areas to optimize the connectivity with the most informative low-level channels [28, 61], and from lowest-level theories according to which skill training induces changes in tuning and other properties of neurons that support memory formation.

This highlights a crucial difference between reverse hierarchy and lowest level theories on the one hand, and the theoretical framework behind double-training studies on the other hand. Lowest-level and reverse hierarchy theories assume a transition from non-retinotopic learning mechanisms during early learning to respectively retinotopic plasticity or selective reweighing of retinotopic inputs in late, asymptotic learning. This leads to the prediction that the smaller performance increments during late learning should be more related to specificity than the larger performance increments during earlier learning. The double-training theoretical framework assumes high-level and non-retinotopic sources of learning in which the presence of specificity depends on task specific or contextual manipulations that can enable or disable generalization [21]. Essentially, the factors that set the amount of specificity are not directly linked with the total magnitude of learning. Moreover, this theoretical view does not provide a basis for a stronger relationship between specificity and the magnitude of performance gains in late asymptotic compared to earlier learning stages.

In addition, the lowest-level theory predicts different spatial gradients of generalization compared to read-out/reverse hierarchy and double training theoretical frameworks. We reasoned that if specificity/generalization were entirely due to processes in non-retinotopic high-level areas, and would not involve any plasticity in retinotopic areas, then expertise should spread equally in the visual field in all directions around a peripheral trained location. Alternatively, if retinotopic areas, which are characterized by cortical magnification of central vision [62], contributed to specificity, then generalization in the visual field from a trained location should be greater towards the periphery than towards the center of vision. Plasticity in horizontal connections [63, 64] may be instrumental in transferring training-induced tuning or signal processing changes from directly stimulated neurons to non-stimulated neurons in retinotopic areas.

To address these hypotheses, we first administered extensive training in Gabor orientation discrimination at a peripheral location, and then tested the magnitude of position specificity by comparing performance in the trained position to performance in neighboring positions of equal polar angle within a visual field quadrant. We also tested orientation specificity in the trained (and other) locations. Secondly, we aimed to derive information from the gradient of generalization among neighboring positions placed both closer to the fovea than the trained location and more peripherally along the same polar angle line. We had expected an asymmetric gradient of generalization, larger towards the periphery than towards central vision, in line with cortical magnification in early visual cortex. Thirdly, we wished to test whether specificity could be predicted from the extent of learning, to gain insight into mechanisms contributing to specificity. We were especially interested in determining whether progress in early or late parts of learning was more strongly correlated with specificity. Note that we were not interested in maximizing generalization, but rather in inducing specificity and then testing hypotheses about its determinants.

## 2 Material and methods

### 2.1 Participants

Ten participants (mean age 25.51, *SD* 5.62, 6 female) naïve to the purpose of the study participated in the main experiment involving orientation discrimination training followed by tests of position and orientation specificity (main experiment). Additionally, ten naïve participants (mean age 21.38, *SD* 2.34, 7 female) were recruited for testing the effect of eccentricity and spatial frequency on orientation discrimination performance (control experiment). All participants had normal or corrected-to normal visual acuity. Informed, written and verbal consent was obtained according to the Helsinki Declaration, after full information about all procedures and the right to withdraw participation at any time. All procedures were approved by the local Ethical Committee of the Faculty of Psychology and Neuroscience (ECP). For their participation in the study, participants received monetary reward, credits to fulfill course requirements, or a combination of both.

### 2.2 Task, Stimuli, and Apparatus

**Fig 1:**
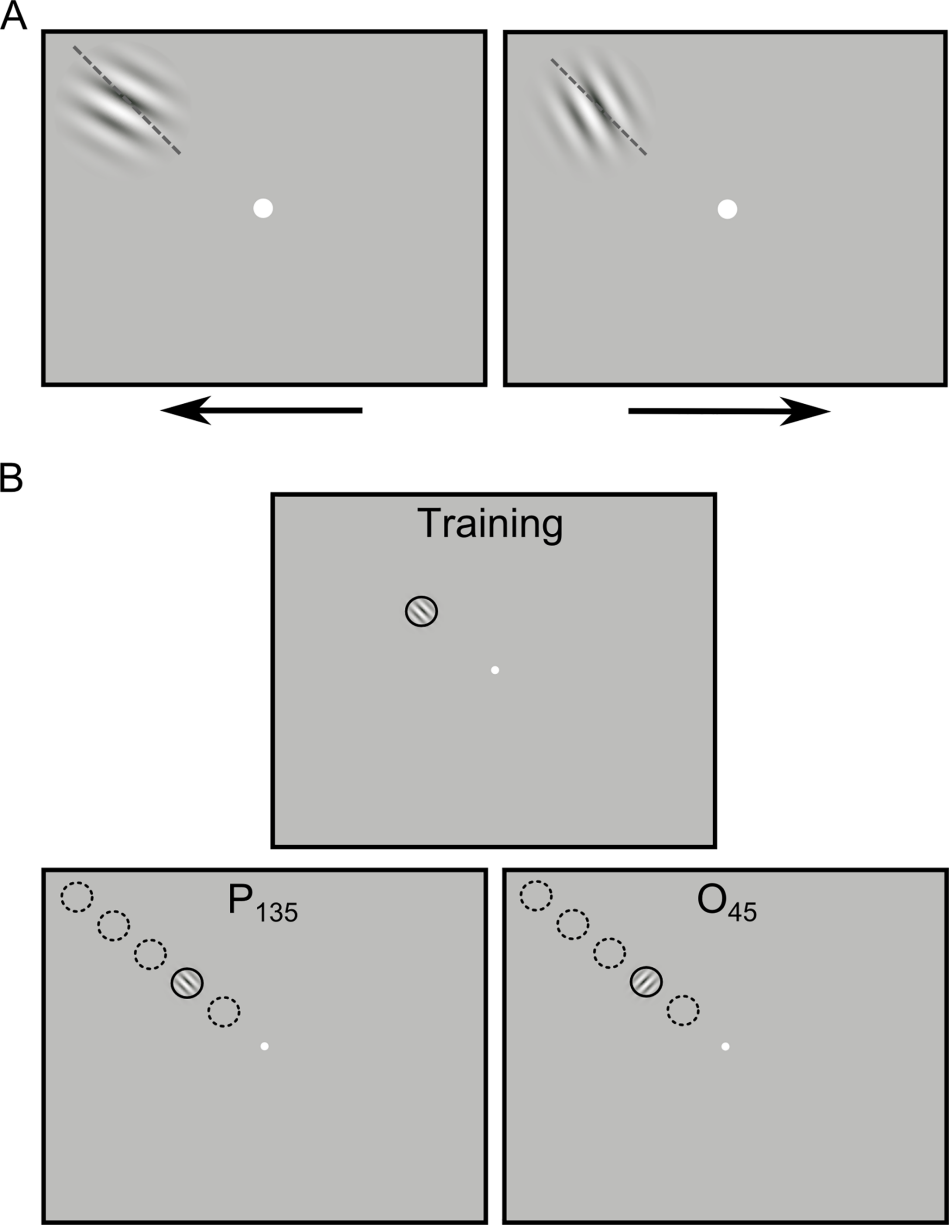
Orientation discrimination task and experimental design. **A**, Stimuli and task. Thresholds were determined using an identification design, with left key responses for counterclockwise orientation offsets from a 135° reference (dashed line, not shown to participants), and right key responses for clockwise orientation offsets. Here, stimuli are enlarged for illustration purposes. **B**, Training phase (top) and the two experimental conditions of the testing phase (bottom), one testing position specificity (P_135_) and the other testing orientation specificity (O_45_). Solid circles represent the position (6° eccentricity) at which training took place. Dashed circles indicate the positions at which specificity was tested: 3°, 9°, 12°, and 15° eccentricity. Dashed and solid circles were not present in actual display and are shown here only for illustration purposes. In the P_135_ and O_45_ conditions, a Gabor stimulus is shown for illustration in the position trained at the 135° reference orientation. Here, the Gabor stimuli are shown to scale.

Participants performed a forced-choice orientation discrimination task with two response options in which participants had to compare a single stimulus to an implicit oblique reference orientation. They indicated the direction of the orientation offset of a Gabor stimulus from the oblique reference by pressing either the left or right arrow key for counterclockwise and clockwise rotations respectively (Fig 1A). In all experiments, Gabor patches around the 135° or 45° reference were used as stimuli (2.37 cycles/degree spatial frequency, 50% Michelson contrast, 0.5° standard deviation, 4 different phases: 0°, 45°, 90°, and 135°, 3° diameter, 500ms duration). The Gabor stimulus was presented at eccentricities of 3°, 6°, 9°, 12°, or 15°, and its average luminance was 56 cd/m^2^, which was equal to the luminance of the background. The most foveal (3°) and peripheral positions (15°) were chosen such that they were approximately equidistant in V1 in terms of cortical distance [62] from the trained position. The stimuli were presented in the upper or lower left or right hemifield along respectively a 135° or 45° iso-polar line. In all experiments, the fixation dot was a small, white disc (0.2°). Participants were placed in a dimly lit room; their head was supported by a chin and head rest keeping eye-screen distance constant at 57 cm. Visual stimuli were displayed on a 19” Samsung SyncMaster 940BF LCD monitor (Samsung, Seoul, South Korea; 60Hz refresh rate, 1280x1024 resolution). The screen was covered by a gray mask with an oval aperture so that the screen borders were not visible to participants and thus could not be used as reference for the orientation discrimination task (the center of the Gabor stimuli was always at least 18.5° from the exterior border of the mask). Fixation control was monitored with a Viewpoint Eyetracker v.2.8.3 (Arrington Research, Inc., Scottsdale, Arizona, USA; 60Hz sampling rate). Stimulus presentation and response recording was performed by Cortex v.5.9.6 (NIH freeware for psychophysical and neurophysiological experimentation). Since the screen dimensions did not permit the presentation of a stimulus at 15° eccentricity while having the fixation spot in the middle of the screen, the screen in that condition was shifted 7cm up or down and 7cm to the left or right (depending on the tested visual field quadrant). The fixation spot was moved oppositely, keeping it in the center of vision. Both the main and control experiment were done with a gaze-contingent display in which trials only started after a period of accurate fixation within 1.5° from the fixation point. Trials were aborted if the participant’s gaze fell outside that specified range.

### 2.3 Training and generalization testing protocols

The experiment consisted of a training protocol followed by a phase of generalization (specificity) testing. Importantly, at the beginning of training, we did not include a pre-test at the transfer positions in the light of experiments showing that even very few trials may lead to substantial position generalization [60]. The complete training protocol comprised a *training scheme* for the trained position in a single quadrant of interest (see Fig 1) as well as various levels of training in other quadrants, to which we refer as the *experimental context* (see Table 1).

**Table 1:** Training schemes.

P	sessions (thresholds)	trained quadrant	aligned with polar angle and fixation dot	delay training- transfer test	delay training -refresher	delay refresher—transfer test	number of refresher sessions	number of refresher staircases	experimental context
1	15 (4)	right upper	during orientation specificity test	32 days	na	na	na	na	1Q-3R
2	10 (8)	left upper	during training	1 day	na	na	na	na	0Q
3	10 (8)	left upper	during training	5 days	na	na	na	na	0Q
4	10 (8)	right upper	during orientation specificity test	32 days	na	na	na	na	0Q
5	15 (4)	left upper	during training	5 days	na	na	na	na	3Q-1R
6	15 (4)	left upper	during training	31 days	na	na	na	na	1Q-3R
7	15 (4)	right upper	during orientation specificity test	11 days	na	na	na	na	3Q-1R
8	15 (4) & refresher	left lower	during orientation specificity test	97 days	90 days	7 days	3	26	1Q-3R
9	15 (4) & refresher	left upper	during training	370 days	367 days	3 days	3	35	2Q-1R & 1Q-3R
10	15 (4)& refresher	right upper	during orientation specificity test	389 days	388 days	1 day	3	39	2Q-1R & 1Q-3R

**Detailed differences in training schemes and experimental context among participants.** The first 9 columns show details about *training schemes*. From left to right, columns show (1) participant number (P), (2) numbers of daily sessions and number of thresholds per session, (3) quadrant used for training (and transfer testing), and (4) whether reference orientation was aligned with polar angle and fixation dot. The next columns show (5) the delay between the end of training and the beginning of transfer testing, and (6) when applicable (na = non-applicable) the delay between the end of training and start of refresher training, (7) the delay between end of refresher training and the beginning of transfer testing as well as (8) the number of refresher sessions and (9) refresher staircases. The study focused on training and generalization within a single quadrant of the visual field (right upper or left upper or lower quadrant). In this quadrant of interest, participants underwent extensive training at a single reference orientation (135°) after which generalization was tested along a set of equi-polar positions within the same quadrant. For the training within this quadrant of interest, participants underwent slightly different *training schemes*. Three groups can be distinguished. Firstly, three participants (P2, 3, 4) were trained only in a single quadrant and underwent 10 sessions with 8 staircases each, immediately followed by transfer tests. Secondly, four participants (P1, 5, 6, 7) trained for 15 sessions with 4 staircases in the quadrant of interest for the present study, again closely followed by transfer testing. Thirdly, three participants (P8, 9, 10) were trained for 15 sessions with 4 staircases. Here, there was a long delay in inviting the participants for the generalization tests, which therefore were preceded by refresher training sessions. In the second and third group, the data in the quadrant of interest for the present study were collected in the context of other experiments performed in other quadrants, falling outside the focus of interest of the present study.

Column 10 summarizes the *experimental contexts* in which the data for the present paper were collected. With the term experimental context, we refer to the presence or absence of threshold measurements obtained in other studies outside the quadrant of interest for the present study. Four groups can be distinguished, marked by 4 codes in column 10, indicating training outside the quadrant of interest. Each code indicates in how many extra quadrants (Q) and for how many references in these quadrants (R) training was obtained. Firstly, three participants (P2, 3, 4) had trained just for the present experiment in a single quadrant, without training in other quadrants (context 0Q; zero extra quadrants). Secondly, in three participants (P1, 6, 8) in which training was performed in one extra quadrant with three references (context 1Q-3R). Thirdly, two participants (P5, 7) underwent training with a single reference orientation in three additional quadrants with a single reference orientation (3Q-1R), as well as subliminal exposure in the lower two quadrants. The training outside the quadrant of interest in the second and third group occurred in the same daily sessions during which the training in our quadrant of interest was carried out. Finally, two participants (P9, 10) had participated in training at a single reference in two extra quadrants, and with three references in a third extra quadrant (2Q-1R & 1Q-3R), prior to training in the quadrant of interest of the present study. For each extra reference orientation, data were collected in 15 daily sessions composed of 4 staircase measurements, and these data were collected over one or more months. Statistical analysis (see Results) has demonstrated that neither training scheme nor experimental context had an effect on the thresholds obtained in the quadrant of interest in the present study or the specificity indices.

The *training scheme* consisted of a phase comprising extensive daily training, which resulted in asymptotic performance levels in a trained position, and was followed by a phase of generalization (specificity) testing. Over the course of 10–15 daily sessions, participants performed the orientation discrimination task (Fig 1A) on a 135° oblique stimulus in an upper quadrant at 6° eccentricity (Fig 1B). A training session consisted of one or two blocks of four staircase measurements (mean 88.55 trials/staircase) determining 84% correct orientation difference thresholds (see Data and Statistical analyses). The complete training scheme led to learning curves composed of at least 60 staircases.

There were some *inter-individual differences in training scheme* related to the stimulus, the organization of training, and the time interval between training and generalization testing (Table 1). Seven participants were trained for fifteen sessions with four staircases per session (60 thresholds), whereas three more participants (P2-4) were trained for 10 sessions with 8 staircases per session (total of 80 thresholds). In three of the participants (P8-10) with 15 sessions, training was supplemented with 26–39 refresher staircases (see Results section). Refresher sessions were aimed to counter potential forgetting in participants who were invited to perform the generalization testing several months after completion of training. The time delay between end of learning and refresher sessions was 4, 10 and 10 months in P8, P9 and P10, respectively. Generalization testing immediately followed the refresher sessions in P8-10, or the end of learning in the seven other participants.

There were also *differences among participants in experimental context;* notably, there were different levels of orientation discrimination training at the same reference orientation of 135° or others, in quadrants other than the quadrant of interest for the present study. The presence of training in extra quadrants as well as at how many references this training took place is summarized in the right-hand column of Table 1 (see legend for details). We demonstrated that neither the variations among participants in experimental context nor training scheme influenced our measures of specificity (see Results, section 3.3 and 3.7).

Because one half of participants was trained (with the 135° reference) in the left upper quadrant, and the other half in the right upper or left lower quadrant, the reference orientation was aligned with polar angle line in the former but not the latter group (Table 1). Alignment could render the fixation spot a potential cue for orientation discrimination. During position specificity testing (with the 135° reference), the alignment condition present during training was maintained. However, during orientation specificity testing (with the 45° reference), the participants in which the reference orientation was aligned during training, underwent the orientation specificity testing at a reference orientation orthogonal to polar angle, whereas in the other group of participants, the opposite was true. Thus, across training and testing of *position* generalization, the alignment condition was constant within participants, half of the group showing alignment, and the other half not. Across training and testing of *orientation* generalization, the alignment condition was switched from alignment to no alignment in one half of the group, and vice versa in the other half. In Results (see Section 3.3), we show that alignment was unlikely to have influenced the magnitude of the thresholds.

The *transfer phase* of the experiment (lower part of Fig 1B) tested the specificity of training for Position at the trained reference orientation (P_135_), and specificity for Orientation using a 45° reference orientation (O_45_), orthogonal to the trained reference. In both conditions, five different eccentric positions were tested: 3°, 6° (trained position), 9°, 12°, and 15°. In the P_135_ condition, eight staircase measurements at the 135° reference were completed per position over two subsequent days, resulting in 40 threshold measurements. In the O_45_ condition, another 8 staircase measurements per position were collected at the 45° reference, yielding another 40 thresholds. On each day, positions were presented in a pseudorandom manner and four staircase measurements in all five test positions were completed. The test for position specificity (P_135_) was carried out first, followed by the test of orientation specificity (O_45_).

Note that for the assessment of position specificity, we did not perform a pre-training versus post-training comparison in the test locations surrounding the trained position. This would have required including some pre-learning threshold measurements in these test locations. We purposefully opted against this experimental design to avoid the possibility that transfer could be due to pre-training measurements in the different test locations (see [60]). Here, we were interested in the amount of transfer to other nearby locations (or the lack thereof referred to as specificity) induced by extended training at a single location, not contaminated by any prior experience in those locations.

In a control experiment we tested the effect of eccentricity on orientation discrimination performance in a group of naïve participants (n = 10). The control participants completed two sessions, in each of which the orientation discrimination task was performed at five different eccentric positions (3°, 6°, 9°, 12°, and 15°). In total, 8 thresholds were obtained at each position. The five positions were arranged along an equi-polar line of 45° (upper right quadrant, stimulus orientation 135°, 5 participants) or 135° (upper left quadrant, stimulus orientation 45°, 5 participants). The order of sessions was counterbalanced across participants as was the order of stimulus positions.

### 2.4 Data collection and statistical analyses

Thresholds were measured using a Wetherill & Levitt [65] staircase tracking 84% correct performance, in which four correct responses in a row led to a decrease in the orientation difference (division by 1.2) and a single incorrect response to an increase (multiplication by 1.2). The staircase was terminated either when 14 reversal points were acquired or 120 trials were completed, whichever came first. The threshold was computed as the geometric mean of the last ten reversal points, where the staircase converged on the orientation difference estimating a 84% correct performance. The first 4 reversal points did not contribute to threshold estimation. Note that the orientation thresholds we report here and in all previous studies testing orientation discrimination from our group [66–71] correspond to the angle between the two orientations, and not to the absolute deviation of the presented orientation and an imaginary reference as done in other studies (e.g., [8, 12]). The latter way of reporting thresholds is based on the idea that participants compare each stimulus with an internal reference. The former way of reporting thresholds is justified by taking into account that the stimulus presentation from the previous trial may help in providing a correct response on the current trial.

For the first training session, the start level of the four staircases was set at an orientation difference of 15°. This starting level in the first session was chosen because it is likely above threshold for most participants (as estimated from a similar study design yielding a mean threshold in the first session of 9.96, SD = 5.76, n = 29 [66]), but not so high that the threshold-level could not be reached within a single staircase. With this starting orientation difference, a level close to the typical first-session threshold (mean 11.44° in our current dataset, SD 6.75, n = 10) can be reached in about two steps, or eight correct responses. In general, well-above threshold examples are thought to facilitate subsequent learning (Eureka effect, [50]). Even if the starting orientation difference in the first session may have been too difficult in some participants, this should then have led to a number of errors quickly leading to large (exemplary) orientation differences. Therefore, the use of the staircase procedure guaranteed the presentation of large orientation differences whenever the starting threshold would have been set too low in some participants.

For all subsequent sessions, including the transfer phase, the average threshold level of the previous session was taken as a starting point. This was done to maximize the number of trials given around threshold level, thereby increasing the extent of asymptotic (late) learning, task difficulty and specificity [51, 54]. Despite the different starting rule between the first session and the later ones, staircases in the first session converged to a level close to the expected 84% correct, as did all other staircases. The last ten reversal points used for threshold calculation corresponded to an 84.9% performance level in the first session, within the 82.5–85.9% range for all additional sessions (averaged over all participants). The length of the staircases of the first session (mean: 93 trials, range: 60–120 trials) was only slightly longer than that for the other sessions (mean: 90 trials, range: 53–120 trials). These observations indicate that all thresholds shown during learning from first to last session reflect a good estimation of the same 84% correct performance level, and hence that the first-session threshold and the faster rate of learning in the beginning part of the learning was not due to a different functioning of the staircases during initial learning.

Following computing the individual staircase thresholds, the natural logarithm was taken to homogenize variance in the sample of participants, after which they were then averaged per condition. Statistical analysis was based on these ln-transformed average thresholds in each condition per participant, with each participant contributing one data point per condition. The term ‘staircase’ is used as shorthand for the staircase threshold measurement procedure, and ‘threshold ‘or ‘staircase threshold’ are used interchangeably to refer to the outcome of the staircase procedure (i.e., a threshold).

To assess position and orientation specificity, data was analyzed with repeated-measures ANOVAs. In cases where the sphericity assumption was violated, the Greenhouse-Geisser correction was applied. For pairwise comparisons, two-sided t-tests were performed (unless mentioned otherwise) and adjusted for multiple comparisons using Bonferroni correction.

In sections 3.1 to 3.3, we used a conventional specificity index based on earlier studies (e.g., [17, 18, 51])). This index was defined as the proportion of total improvement in the training stage that is not transferred to the new position at the point of the transfer test. On average, participants tended to have lower thresholds at the trained position during transfer testing compared to during the last session of training. Similarly to Jeter and colleagues [17] we therefore also calculated the index using not the end of training but the transfer test threshold for the trained position and orientation as reference, referred to as $\bar{O_{te}}$:

$$S\prime =\left({\bar{O_{test}}}_{\left(o,\;e\right)}-{\bar{O_{test}}}_{\left(135{}^{\circ},\;6{}^{\circ}\;ecc\right)}\right)/\left({\bar{O_{train}}}_{begin(135{}^{\circ},\;6{}^{\circ}\;ecc)}-{\bar{O_{test}}}_{\left(135{}^{\circ},\;6{}^{\circ}\;ecc\right)}\right)$$ (1)

where ${\bar{O_{train}}}_{begin(135{}^{\circ},\;6{}^{\circ}\;ecc)}$ and ${\bar{O_{test}}}_{\left(135{}^{\circ},\;6{}^{\circ}\;ecc\right)}$ are the orientation discrimination thresholds for the first training session and transfer test respectively at the trained position and reference orientation, and ${\bar{O_{test}}}_{\left(o,\;e\right)}$ the orientation discrimination threshold during transfer test where o and e indicate the reference orientation and eccentricity. This index, referred to in our paper as the conventional specificity index, can range between 0 and 1, where a value of 0 means no specificity (full transfer), a value of 1 means complete specificity. Partial specificity is assumed when the index differs significantly from 0. To assess the influence of training scheme and experimental context on the conventional specificity indices, it was necessary to calculate the specificity indices using the threshold from the last training session as reference (instead of the transfer test threshold for the trained position and orientation), as otherwise the values for the trained position and orientation become zero by definition and thus cannot show variance.

We also used specificity indices different from the conventional specificity index (in sections 3.5 to 3.7). The reason for this was that we wanted to relate the specificity index to different stages of learning. To do so, a specificity index is required that is different from the index that measures progress during training. In Jeter’s conventional index S’, the denominator reflects the improvement during training, and we would essentially correlate S’ with its own denominator. It is also not clear how to modify Jeter’s index if one wants to correlate progress in early, middle, or late phases of learning with specificity.

With the aim in mind to assess training extent and specificity with different indices derived from non-overlapping data, we quantified position, orientation specificity as well as training-related performance increases, using a Michelson index. The training index quantified the amount of performance increase for the total length of training, or within a specific phase of training and was calculated as:

$$T=\frac{{\bar{O_{train}}}_{begin(135{}^{\circ},\;6{}^{\circ}\;ecc)}-{\bar{O_{train}}}_{end(135{}^{\circ},\;6{}^{\circ}\;ecc)}}{{\bar{O_{train}}}_{begin(135{}^{\circ},\;6{}^{\circ}\;ecc)}+{\bar{O_{train}}}_{end(135{}^{\circ},\;6{}^{\circ}\;ecc)}}$$ (2)

Here, ${\bar{O_{train}}}_{begin(135{}^{\circ},\;6{}^{\circ}\;ecc)}$ and ${\bar{O_{train}}}_{end(135{}^{\circ},\;6{}^{\circ}\;ecc)}$ represent the average of the first and last group of 4 orientation thresholds obtained in a specific training phase at the eccentricity position of 6°. The mean learning curve averaged over all participants was based on 60 individual thresholds. Hence, thresholds used to compute the training index for the whole learning curve compared the average of individual thresholds 1–4 with the average of individual thresholds 57–60. Training indices were also calculated separately for the first, second, and last tertile of the learning curve. Some participants had done more than 60 staircase thresholds because of a different training protocol (P2-4) or additional refresher thresholds (P8-10). Therefore, for these participants, we also calculated the training index T using end thresholds 77–80 in P2-4 and the last 4 refresher thresholds in P8-10 (see Results).

The Michelson-index-based position specificity (PS) index quantified the difference between performance at the trained position and performance at all other test positions and was calculated as:

$$PS=\frac{{\bar{O_{test}}}_{\left(135{}^{\circ},\;other\;ecc\right)}-{\bar{O_{test}}}_{\left(135{}^{\circ},\;6{}^{\circ}\;ecc\right)}}{{\bar{O_{test}}}_{\left(135{}^{\circ},\;other\;ecc\right)}+{\bar{O_{test}}}_{\left(135{}^{\circ},\;6{}^{\circ}\;ecc\right)}}$$ (3)

Here, ${\bar{O_{test}}}_{\left(135{}^{\circ},\;6{}^{\circ}\;ecc\right)}$ represents the orientation threshold from the trained position in condition P_135_ during the transfer phase and ${\bar{O_{test}}}_{\left(135{}^{\circ},\;other\;ecc\right)}$ represents the orientation threshold from either the remaining four positions in the same condition, or the mean of only the two surrounding positions (3° and 9°). Which test positions were included in ${\bar{O_{test}}}_{\left(135{}^{\circ},\;other\;ecc\right)}$ for determining the PS is explicitly indicated in the text.

The Michelson-index-based orientation specificity (OS) index quantified the difference between performance at the trained position for the trained orientation (135°) and the control orientation (45°) and was calculated as:

$$OS=\frac{{\bar{O_{test}}}_{\left(45{}^{\circ},\;6{}^{\circ}\;ecc\right)}-{\bar{O_{test}}}_{\left(135{}^{\circ},\;6{}^{\circ}\;ecc\right)}}{{\bar{O_{test}}}_{\left(45{}^{\circ},\;6{}^{\circ}\;ecc\right)}+{\bar{O_{test}}}_{\left(135{}^{\circ},\;6{}^{\circ}\;ecc\right)}}$$ (4)

Here, ${\bar{O_{test}}}_{\left(45{}^{\circ},\;6{}^{\circ}\;ecc\right)}$ represents the threshold from the untrained orientation in condition O_45_ at the trained position during the transfer phase, and ${\bar{O_{test}}}_{\left(135{}^{\circ},\;6{}^{\circ}\;ecc\right)}$ represents the orientation threshold from the trained orientation in condition P_135_ at the trained position. For all indices, we performed 1.5 Interquartile Range (IQR) criterion outlier detection, to exclude biasing any correlations based on those indices by extreme data points. We used ordinary least square (OLS) regression analysis to predict position and orientation specificity from training indices. Robust regression analysis was additionally used to further test for the contribution of extreme data points.

## 3 Results

### 3.1 Within-quadrant transfer tests after training show significant position specificity and orientation specificity

**Fig 2:**
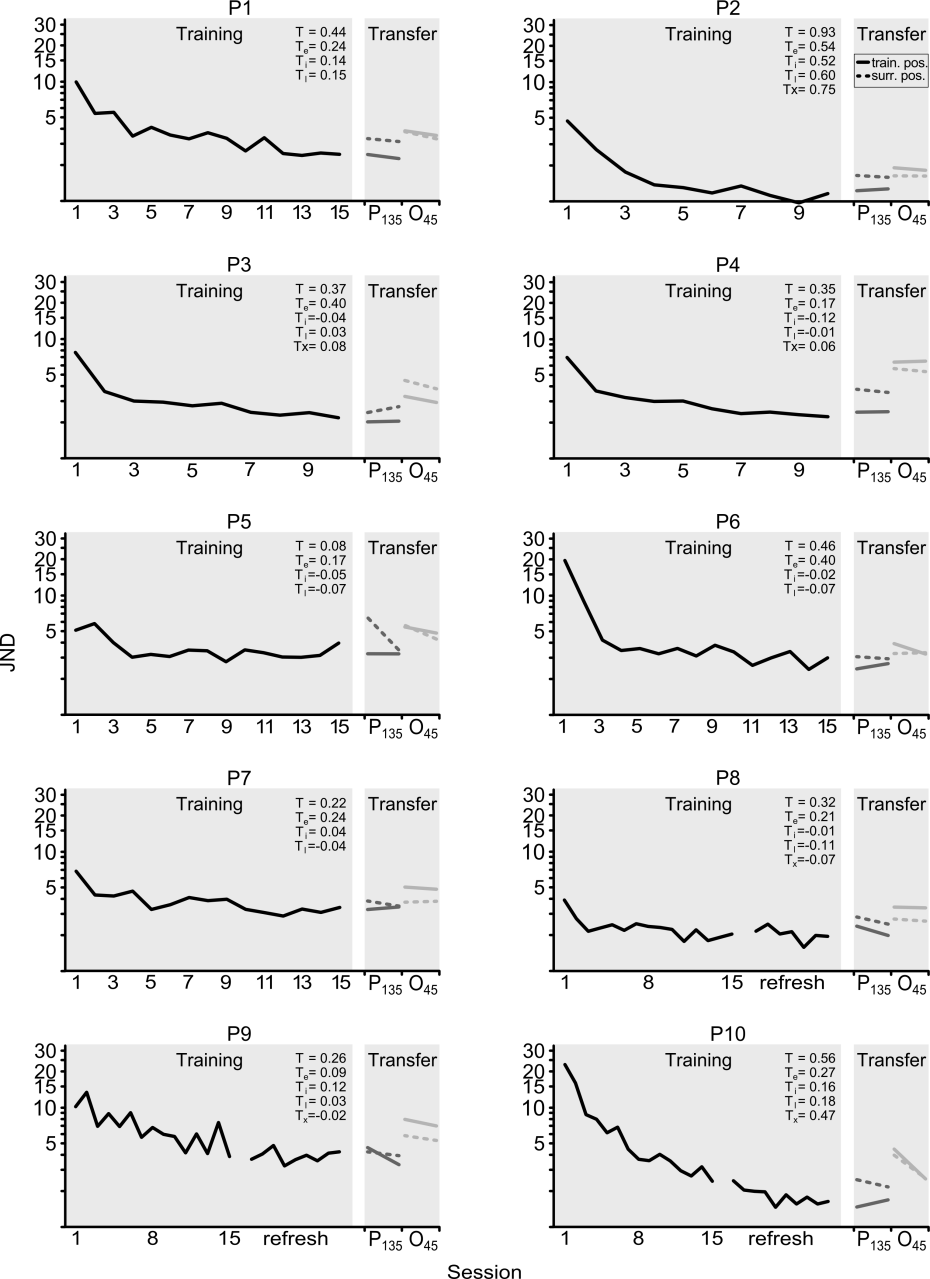
Individual learning curves for all ten participants. Participants completed slightly different training schemes: In participants 1–7, the end of training was followed immediately by the two transfer tests. Participants 8–10 received their training ~6 months prior to generalization testing, and therefore refresher thresholds (26–39 thresholds) were done prior to generalization testing (other details in Table 1). In all participants, the quadrant where training occurred (upper left or upper right quadrant, see Table 1) had never been exposed to any training with visual stimuli. For the quadrant in which training occurred in the present study, the training index T for the entire learning amplitude is shown, as well as the training indices for early learning (Te), intermediate phase learning (Ti), late phase learning (Tl) as well as the ‘extra-late’ phase learning (Tx). During transfer testing, position transfer at the 135° reference orientation (P_135_) and orientation transfer to the 45° reference orientation (O_45_) were done back to back in all participants, with position transfer testing always preceding orientation transfer testing. Overall, the data indicate a difference in the P_135_ condition between thresholds in the standard trained position (dark-grey solid lines) and thresholds pooled over all other positions (dark-grey dashed lines). In the O_45_ condition, overall, there was no analogous difference between thresholds at the 45° reference orientation in the location trained with the 135° reference orientation (light-grey solid lines) and thresholds at the 45° reference pooled over the other locations (light-grey dashed lines). However, using an untrained reference orientation (both light-grey lines) overall seemed more detrimental for the magnitude of thresholds than using an untrained position (dark-grey dashed line).

Fig 2 shows data from individual participants in ten separate panels, showing in each panel training thresholds at the 135° reference orientation on the left, and the transfer test thresholds (position and orientation specificity test) on the right. On the left in each panel, the complete *training phase* is shown for 15 sessions of four thresholds in seven participants, or for 10 sessions of eight thresholds in three other participants (P2-4). In three participants (P8-10), refresher thresholds are shown following the learning curve. These thresholds were collected just prior to generalization testing to counter possible forgetting due to a time delay in inviting these participants for the generalization test (see Methods). Overall, participants showed considerable learning during the training phase at the 135° oblique reference orientation. On the right in each panel, performance in the *transfer-testing phase* is shown by comparing the threshold at the previously trained position (solid lines) with the pooled threshold from all other neighboring generalization-test positions (dashed lines). This comparison was done separately for the P_135_ and O_45_ generalization tests. Both generalization tests consisted of two sessions of 4 thresholds per test position in each participant, as shown in the right-hand part of each figure panel. Overall, the transfer data in the P_135_ condition suggest a trend for test thresholds at the 135° reference orientation to be higher in non-trained positions (dashed dark-grey line) than in the trained position (solid dark-grey line). This difference is not systematically present for test thresholds measured in the O_45_ condition (45° reference orientation, light grey solid and dashed lines). Moreover, thresholds in the O_45_ condition exceeded those in the P_135_ condition for the initially trained position (compare the solid dark-grey and light-grey lines) but also for the other positions (dashed dark-grey and light-grey lines).

**Fig 3:**
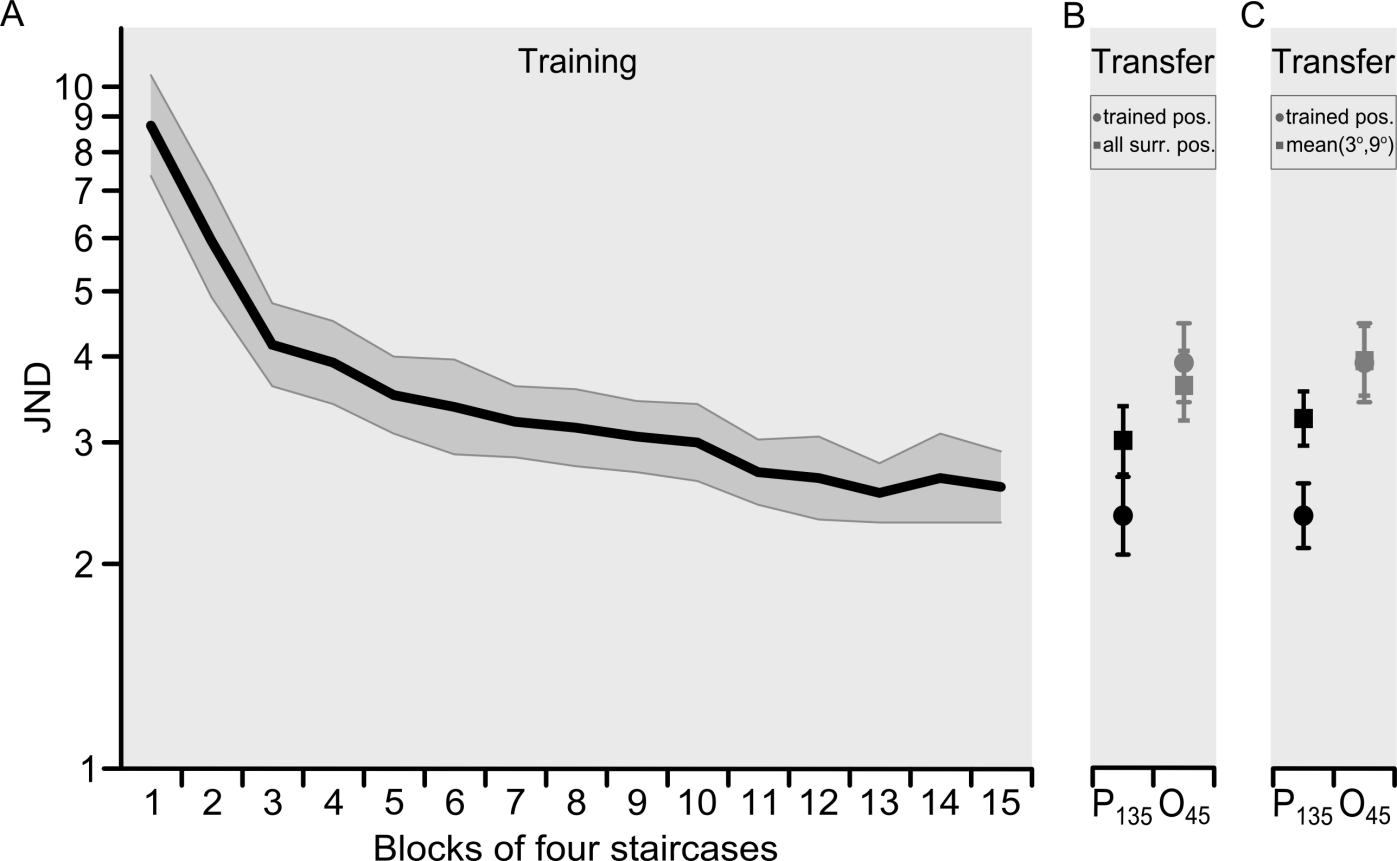
Mean performance during training (***A***) and transfer testing phase (***B***, ***C***). ***A***, Each data point of the learning curve corresponds to the average of ten thresholds (1 per participant), with each participant’s threshold based on four staircase thresholds. In three participants, blocks do not correspond to daily sessions (details in text). ***B*,** The data from the transfer phase (one data point per participant, based on the average of eight staircase thresholds) shows thresholds for 135° (black symbols) and 45° (grey symbols) stimuli conditions at the trained position at 6° eccentricity (circles) and the average of all surrounding generalization test positions–each tested with 8 threshold in all participants (squares). **C,** Same data as in B except that the square symbols only represent the data from the two directly surrounding test positions at 3° and 9° eccentricity. Error bars represent the standard error of the mean. Some participants underwent four staircases in each of 15 sessions (P 1, 5, 6, 7,8, 9,10), whereas others underwent eight staircases in each of 10 sessions (P 2, 3, 4), with session referring to a single testing period in a single day in which either 4 or 8 staircases thresholds were collected. Participants with 15 sessions of 4 staircases had a mean of 90.06 trials per staircase (sd 15.97) (average of 360.24 trials/session) and the participants with 10 sessions of 8 staircases had a mean of 88.04 trials per staircase (sd 14.02) (704.32 trials/session). We wanted to compare and average the learning curves over participants despite the different distribution of numbers of staircase thresholds over daily sessions in the above-referred participants. To do this, we chose here to display average thresholds as a function of blocks of 4 staircases in all participants. In 7 out of 10 participants, blocks correspond to sessions. In the other three participants, each single training session of 8 staircases is split in 2 blocks. This means that for P2-4 data based on the final 20 thresholds are not included here, but their effect in generalization is evaluated in separate analyses (see results in section 3.5 and 3.6).

Fig 3A shows the data averaged over all ten participants. Given the fact that in 3 participants training data were collected in 10 daily sessions of 8 thresholds, and in 15 daily sessions of 4 thresholds in the other participants (left column Table 1), we took the first 60 thresholds of all ten participants and divided them into blocks of four thresholds, resulting in 15 data points for the learning curve in each participant. Henceforth, a ‘block’ refers to a group of four staircase thresholds, as opposed to a session, which refers to all thresholds collected on the same day (i.e., two blocks per session in three participants, and 1 block per session in the others). Limiting the learning curve to 60 thresholds (15 blocks) means that neither the last 20 thresholds of P2-4, nor the refresher thresholds in P8-10 were included in Fig 3A (see also Table 1). Note furthermore that each data point in the learning curve corresponds to ten average thresholds, one per participant, with each data point in an individual representing the average of four individual staircase thresholds. In the transfer phase, each data point represents the average of ten participants, each contributing the average threshold of eight staircase thresholds. Analysis showed that there was no difference between the first and last four thresholds obtained during transfer testing for any of the combinations of position (3°, 6°, 9°, 12°, 15°) and condition (P_135_, O_45_) (all *p* > .242) , which is why we here show transfer thresholds that per participant are based on 8 staircase thresholds.

Note that the thresholds in the test positions were averaged in two ways, either including all test positions surrounding the trained position (Fig 3B), or only including the two test positions next to the trained position (Fig 3C). The latter representation of the data will be discussed in section *3*.*4 Position and orientation specificity based on immediately surrounding positions*. Taken together, when compared to the amplitude of learning shown in Fig 3A, the data show large within-quadrant generalization of orientation discrimination performance. Nevertheless Fig 3B (and similarly, Fig 3C), also demonstrates a difference in the P_135_ condition between thresholds in the standard trained position (black circle) and in the test positions (black square), in line with significant position specificity. An analogous difference was not present for the O_45_ condition (grey symbols). In addition, compared with thresholds at the 135° reference in the trained position (black circle), thresholds at the untrained 45° reference orientation in the same position (grey circle) showed a pronounced increase, suggesting substantial specificity for reference orientation. The increased thresholds at the 45° reference orientation in test positions surrounding the trained position (grey black square) also support specificity.

To statistically test the data obtained during generalization testing, we used a repeated measures ANOVA on the transfer test data as shown in Fig 3B. This analysis revealed a significant main effect of condition (P_135_, O_45_) (*F*_(1, 9)_ = 41.339; *p* < .001), a non-significant main effect of test position (trained, others) (*F*_(1, 9)_ = 5.464; *p* = .044), and a significant interaction between these two factors (*F*_(1, 9)_ = 31.792; *p* < .001). As the interaction indicated that the position effect differed between the two reference orientations, a repeated measures ANOVA on position (trained, others) was performed for condition P_135_ and O_45_ separately. For the P_135_ condition there was a highly significant effect of position (*F*_(1, 9)_ = 38.898; *p* < .001), with the orientation discrimination test threshold at the trained position (Fig 3B, black circle) being lower than the average threshold of all other test positions (Fig 3B, black square). In the O_45_ condition (grey symbols), in which orientation discrimination was tested with the untrained 45° reference orientation, the repeated measure ANOVA resulted in no significant main effect of position (*F*_(1, 9)_ = 1.747; *p* = .219). This indicates that the emergence of position specificity at the end of learning was specific for the trained reference orientation. Interestingly, overall, the thresholds in the O_45_ conditions in Fig 3 were considerably higher than the thresholds for asymptotic learning of the trained 135° reference orientation, returning roughly to the performance level observed in the fourth training session. This suggests a relatively large amount of orientation specificity.

Next, we wished to control for potential influences of training scheme and experimental context on specificity. To that aim, we repeated the statistical comparison of thresholds at the 135° reference between trained and test positions (assessing position specificity), and the comparison of thresholds when switching from the 135° reference to the 45° reference orientation in the position trained with the 135° reference. However, this time we used training scheme and experimental context as covariates. In neither analysis were these covariates significant (all *p* > .798). When comparing the size of the position and orientation effect over participants, the data revealed larger orientation specificity indices than position specificity indices (*t*_*(9)*_ = -4.203, *p* = .016). To assess the precise magnitude of specificity relative to the amount of learning during training, we calculated the conventional position specificity index for the average of all non-trained test positions and the conventional orientation specificity index across all eccentricities. This showed that despite a large amount of generalization of performance within a quadrant there was partial position specificity (S’ = 0.251, *p* = .007) and partial orientation specificity (S’ = .513, *p* = .001). These values show that after switching to a different position, 25.1% of original learning was lost, whereas after switching reference orientation, 51.3% of original learning was lost. The conventional specificity indices thus showed that orientation specificity was twofold larger than position specificity, which was confirmed by a statistically significant difference between the two (*t*_*(9)*_ = 3.839, *p* = .004). This result however is linked with our experimental design, and should not be taken as evidence that orientation specificity is generally larger than position specificity. In the present experiment, the orientation difference during generalization testing was maximized (testing at an orthogonal reference orientation), whereas the position difference over which generalization was tested was minimized (limiting ourselves to close locations within a quadrant).

Furthermore, we noticed a remarkable trend in the data related to orientation specificity. In the non-trained test positions that were tested at the 45° and the 135° reference orientation (compare square symbols), there was a near-significant trend for orientation specificity (*t*_*(9)*_ = -3.446, *p* = .051). This suggests that there can be orientation specificity in locations that have not been exposed to any training, but at threshold levels that exceed the level attained after complete training. For completeness, we also report that for the untrained orientation (45°) the difference in thresholds between the trained and the untrained, surrounding positions was not significant (compare grey symbols: *t*_*(9)*_ = 1.322, *p* = >.999), demonstrating that position specificity did not extend to the untrained orientation. Accordingly, the difference in threshold between trained and non-trained locations for the 135° reference orientation was significantly larger than the difference in thresholds between trained and non-trained locations for the 45° reference orientation (*t*_*(9)*_ = 5.651, *p* = .002). This supports the idea that full training at the 135° reference orientation at the 6° eccentricity position creates a form of expertise that is linked to both the trained position and the trained reference orientation. Overall, the data also show that the variation in thresholds due to position and orientation specificity was relatively small compared to the decrease in thresholds from the beginning to the end of learning. However, analysis of thresholds as well as the use of the S’ specificity index supported the presence of significant specificity. Hence, the data indicate that much, but not all, of the performance benefit of learning can generalize over position and orientation for test positions in the same quadrant as the training location. Accordingly, testing the trained 135° reference orientation at a neighboring transfer position yields a threshold that corresponds closely to the threshold in the trained position in session 8, whereas testing an orthogonal (45°) reference orientation in the trained position yields a threshold closely corresponding to the threshold in session 4. The loss of the late part of learning during generalization testing suggested that performance gains in later parts of learning may be preferentially related to specificity (see *3*.*5 Position specificity is related to the extent of late learning and 3*.*6 Orientation specificity is related to the extent of late learning*).

### 3.2 Does asymmetric transfer suggest retinotopic contributions to position specificity?

**Fig 4:**
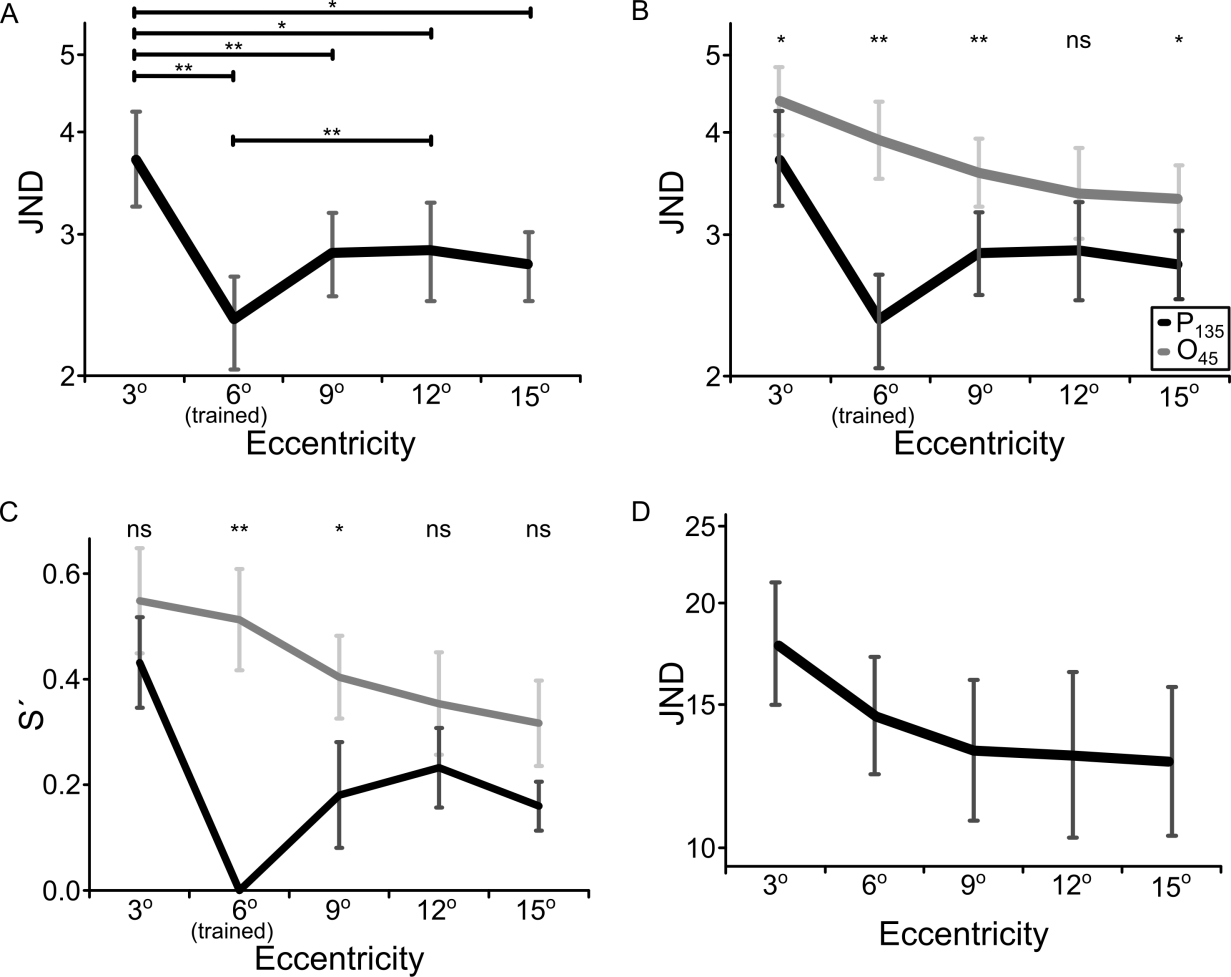
Orientation discrimination thresholds as a function of stimulus position eccentricity. ***A*,** Transfer test with trained reference orientation. Trained participants completed 15 sessions of training at a reference orientation of 135° and subsequently performed the transfer test at that reference (black line: P_135_) (* *p* < .05, ** *p* < .01). ***B*,** Transfer test with an orientation reference orthogonal to that used during training. The same participants whose data is shown in (A) for P_135_ performed the transfer test at the non-trained reference orientations of 45° (grey line: O_45_). Data from the P_135_ transfer condition are copied for comparison (black line). Asterisks indicate significance of threshold differences (* *p* < .05, ** *p* < .01). ***C***, Specificity index for the P_135_ and O_45_ condition for all eccentricities. The index used the 6° eccentricity condition for the trained orientation as a reference to assess the amount of specificity (for details see Methods). Partial specificity was found for all eccentricities and orientations except at 9° in the P_135_ condition. Asterisks indicate significance of differences in S’ (* *p* < .05, ** *p* < .01) for position (P_135_) and orientation (O_45_). ***D*,** Control experiment testing effect of eccentricity on orientation thresholds in untrained participants. Naïve control participants without any prior training performed orientation discrimination at either a 135° or a 45° reference orientation (with quadrant choices avoiding alignment of reference orientation with the polar line) at five different stimulus position eccentricities. Error bars show standard error.

We had hypothesized that due to cortical magnification in retinotopic areas, there would be less transfer from the trained position towards the fovea than towards the periphery for matched distances in the visual field. To test this asymmetric transfer hypothesis, we performed a repeated measures ANOVA with thresholds of each of the five eccentric positions (3°, 6°, 9°, 12° and 15°, see Fig 4A) as a within-subjects factor. The results showed that the main effect of stimulus position was highly significant (*F*_(4, 36)_ = 17.162; *p* < .001). Pairwise comparisons (Fig 4A) showed that thresholds at the most central position differed from thresholds at all other eccentricities (3° vs. 6°—*t*_*(9)*_ = 8.162, *p* = .001; 3° vs. 9°—*t*_*(9)*_ = 5.459, *p* = .004; 3° vs. 12°—*t*_*(9)*_ = 4.335, *p* = .019; 3° vs. 15°—*t*_*(9)*_ = 4.611, *p* = .013). The trained position also differed significantly from the 12° position (6° vs. 12°: *t*_*(9)*_ = -5.595, *p* = .003). The remaining comparisons between positions did not differ significantly from each other (6° vs. 9°: *t*_*(9)*_ = -2.714, *p* = .239; 6° vs. 15°: *t*_*(9)*_ = -3.453, *p* = .072; 9° vs. 12°: *t*_*(9)*_ = 0.109, *p* > .999; 9° vs. 15°: *t*_*(9)*_ = 0.547 *p* > .999; 12° vs. 15°: *t*_*(9)*_ = 0.819, *p* > .999). Hence, the data seem to suggest that the expertise gained at the 6° trained position generalized more towards peripheral positions than to the more central position. For example, there was a larger threshold increase from the trained position (6°) to the most central 3° position compared to the threshold increase at the 9° position. This may suggest a contribution of retinotopic mechanisms to position specificity, with less transfer towards the foveal position (corresponding to larger cortical distance) and more transfer towards a peripheral position (over the same visual field distance but a smaller cortical distance).

This interpretation can be questioned however based on thresholds obtained at the untrained 45° reference orientation in all test positions. Considering the data for the 45° reference orientation alone (grey line in Fig 4B), the data simply suggest that orientation discrimination improved with eccentricity (repeated measures ANOVA, *F*_(4, 36)_ = 4.389, *p* = .005). To test whether the advantage with increasing eccentricity was dependent on reference orientation, we performed a repeated measures ANOVA combining the data from the two reference orientations. The analysis included reference orientation (P_135_, O_45_) and position (3°, 6°, 9°, 12° and 15°) as within-subject factors and revealed a significant main effect of reference orientation (*F*_(1, 9)_ = 22.981; *p* = .001), a significant main effect of eccentric position (*F*_(4, 36)_ = 11.429; *p* < .001) and a significant interaction (*F*_(4, 36)_ = 8.337; *p* < .001). A comparison of orientation discrimination thresholds for reference orientations 45° and 135° per eccentricity revealed that all thresholds except at 12° were significantly different (3°: *p* = .036; 6°: *p* < .001; 9°: *p* = .009; 12°: *p* = 0.057; 15°: *p* = .025; see asterisks in Fig 4B).

Note that the interaction between reference orientation and eccentricity was mainly due to the difference between thresholds at the 6° position, which confirms the strong orientation specificity at that position. When excluding that position, a repeated measures ANOVA using reference orientation (E_135_, C_45_) and position (3°, 9°, 12° and 15°) as within-subjects factors confirmed the significant main effects of reference orientation (*F*_(1, 9)_ = 11.742; *p* = .008) and eccentricity (*F*_(3, 27)_ = 13.242; *p* < .001), but did not show a significant interaction (*F*_(3, 27)_ = 0.371; *p* = .774). Thus, orientation discrimination performance at both the 45° and the 135° reference was better at higher eccentricities. This suggests an advantage in orientation discrimination at higher eccentricities that could be due either the use of a low-frequency Gabor stimulus, or to a learning effect. Therefore, the data do not support that the within-quadrant gradient in transfer would reflect a contribution of cortical magnification of central vision in retinotopic areas.

A further argument against a contribution of cortical magnification to the within-quadrant transfer gradient over positions of skill at the 135° reference orientation is related to the difference in thresholds at the 15° and 3° positions. These two positions had been chosen to approximately match in cortical distance in V1 and V2 from the trained position (3°), and hence we had expected that the thresholds of these two positions would be approximately the same. Contrary to these expectations, the thresholds differed significantly at these two positions (*p* = 0.013) (see Fig 4A).

To further assess the magnitude of position specificity per eccentricity at the 135° reference orientation, we calculated the conventional specificity index S’ (Fig 4C, black line). Partial specificity was found for 3° (S’_3°_ = .431, *p* = .001), 12° (S’_12°_ = .233, *p* = .016), and 15° (S’_15°_ = .160, *p* = .011), however at 9° there was no evidence for partial specificity (S’_9°_ = .181, *p* = .114). Thus, moving 3° towards the fovea from the trained position resulted in significant specificity, whereas moving 3° towards the periphery from the trained position did not result in significant specificity, possibly supporting a retinotopic mechanism for position specificity. However, specificity expressed by S’ was significantly smaller at 15° compared to 3° of eccentricity (*t*_*(9)*_ = 3.751, *p* = .005), which was not expected. Moreover, an eccentricity-dependent gradient in S’ was present at the 45° reference orientation (Fig 4C, grey line), similar to that observed at the 135° reference orientation (when excluding the 6° position; Fig 4C, black line). This is in line with the interpretation that for the chosen stimulus there was a general orientation discrimination performance advantage at the largest eccentricities compared to the smallest one, thus questioning the interpretation that cortical magnification in retinotopic areas would drive the observed spatial pattern of generalization.

To evaluate whether the trend towards better performance in the peripheral test positions reflected an advantage of low spatial frequency stimuli at higher eccentricities, or perhaps a learning-related effect, we conducted a control experiment in ten naive participants without prior orientation discrimination training. They performed orientation discrimination with the same Gabor stimulus (2.37 cycles/degree) along eccentricities ranging from 3° to 15° along an equi-polar line in a single visual field quadrant (see Methods for details). The data of the untrained participants also showed decreased thresholds for higher eccentricities (Fig 4D). This was confirmed in a repeated measures ANOVA with eccentricity (3°, 6°, 9°, 12°, 15°) as within-subjects factor, which showed a significant main effect (*F*_*(2*.*214*, *19*.*922)*_ = 5.118, *p* = .014). Since participants performed eight staircases in each position spread over two sessions (four per session), we wished to test whether this high eccentricity advantage disappeared in the second session. Additional analysis including session (session 1 and 2) as a second within-subject factor revealed decreased orientation discrimination thresholds with increasing eccentricity to the same extent in sessions 1 and 2, as the interaction between session and position was not significant (*F*_*(4*, *36)*_ = 1.053, *p* = .394). We also checked for session and position order effects as one could hypothesize that participants improved over time and thus were performing better at positions that were trained later in a session. There was a significant session effect (*F*_(1, 9)_ = 13.088, *p* = .006), but the position order effect (*F*_(4, 36)_ = 1.354, *p* = .269) and the interaction (*F*_(4, 36)_ = 1.444, *p* = .239) were non-significant. Participants thus improved across sessions but in both sessions performance was better for more peripheral stimulus positions irrespective of the order in which positions were tested. Taken together, the data indicate that the better performance in the orientation discrimination task for more peripheral stimulus positions was due to an interaction between stimulus characteristics and eccentricity and not to a learning effect.

It is noteworthy that the data in Fig 4B and 4C also confirm significant orientation specificity. In the experimental design used, OS tended to be larger than PS at all but one position used (as indicated by asterisks in Fig 4B on top of corresponding threshold measurements), which is in line with the significant main effect of reference orientation in ANOVA on thresholds in Fig 4B (details in paragraphs above describing the analysis of Fig 4B). In addition, computing the conventional orientation specificity index separately for all eccentricities, partial but significant orientation specificity was found for 3° (S’_3°_ = .547, *p* < .001), 6°(S’_6°_ = .513, *p* = .001), 9° (S’_9°_ = .403, *p* = .001), 12° (S’_12°_ = .354, *p* = .006), and 15° (S’_15°_ = .316, *p* = .004) (see also Fig 4C, grey line). In the experimental design we used, S’ tended to be larger for orientation than for position specificity (see asterisks in Fig 3C). The differences in S’ for 3° (ΔS’_3°_ = .116, *p* = .062), 6°(ΔS’_6°_ = .513, *p* = .001), 9° (ΔS’_9°_ = .222, *p* = .011), 12° (ΔS’_12°_ = .089, *p* = .006), and 15° (ΔS’_15°_ = .157, *p* = .054) were significant for 6° and 9° but failed to reach significance for the remaining eccentricities. Taken together, the data suggest that with training, participants acquired a skill with a significant degree of specificity for position and orientation at the trained 6° position. In addition, at the trained reference orientation, there was a tendency for an asymmetric spread of orientation discrimination skill from the trained position to the immediate foveal and peripheral neighbors, with higher thresholds at the more foveal than at the more peripheral position. However, in the light of the described control analyses, this cannot be convincingly interpreted as support for a contribution to position specificity from mechanisms in retinotopic areas.

It is possible that specificity would have been higher if only initial thresholds in each test position with the trained reference orientation, or with the orthogonal reference orientation, would have been included in the specificity index S’. To assess the influence of within-session averaging of performance, we ran a repeated-measures ANOVA with reference orientation (P_135_, O_45_) and position (3°, 6°, 9°, 12° and 15°) and threshold measurements (1–8) as within-subject factors. While the main effect of reference orientation (*p* = .007) and position (*p* = .002) as well as their interaction were significant (*p* = .004), neither the main effect of threshold measurements (*p* = .059) nor any of the interactions with threshold measurements turned out significant (all *p* > .195). This indicates that any threshold differences over the 8 staircase measurements per position and reference orientation were too small to affect our results in a meaningful way.

### 3.3 Details of training scheme and differences in experimental context among participants do not affect within-quadrant transfer

To test effects of slight differences in training scheme as well as differences in experimental context on generalization, we performed a repeated measures ANOVA with condition (P_135_, O_45_) and eccentricity (3°, 6°, 9°, 12°, 15°) as within-subject factors (see Fig 4D) and moreover training scheme (15x4, 10x8, 15x4-Refresh) and experimental context (4 conditions, see Table 1) as between subject factor. Neither the between-subject effects for training scheme (*p* = .729) and experimental context (*p* = .709) nor any of the interactions with training scheme (condition x training scheme: *p* = .897; eccentricity x training scheme: *p* = .705; condition x eccentricity x training scheme: *p* = .964) or experimental context (condition x experimental context: *p* = .519; eccentricity x experimental context: *p* = .173; condition x eccentricity x experimental context: *p* = .876) were significant. So, we found no effect of training scheme or experimental context on thresholds, indicating these factors cannot be used as an alternative explanation for differences among participants in thresholds.

To exclude that training scheme and experimental context affected the conventional specificity indices we calculated the specificity indices using the threshold from the last training session as reference. We then performed a repeated measures ANOVA on these indices with condition (P_135_, O_45_) and eccentricity (3°, 6°, 9°, 12°, 15°) as within-subject factors (see Fig 4D) and moreover training scheme (15x4, 10x8, 15x4-Refresh) and experimental context (4 conditions, see Table 1) as between subject factor. Again, neither the between-subject effects for training scheme (*p* = .392) and experimental context (*p* = .476) nor any of the interactions with training scheme (condition x training scheme: *p* = .888; eccentricity x training scheme: *p* = .762; condition x eccentricity x training scheme: *p* = .823) or experimental context (condition x experimental context: *p* = .984; eccentricity x experimental context: *p* = .996; condition x eccentricity x experimental context: *p* = .983) were significant.

To test whether the pattern of position and orientation generalization reported in Section 3.2 was influenced by alignment cues (See Methods and Table 1), we also performed additional analysis. When re-running the repeated measures ANOVA with reference orientation (P_135_, O_45_) and position (3°, 6°, 9°, 12° and 15°) as within-subject factors separately for the two halves of participants (according to whether or not alignment was present during training), we replicated in both cases the two main effects and the interaction (eccentricity effect: *p* = .012 unaligned, *p* = .001 aligned; orientation effect: *p* = .029 unaligned, *p* = .037 aligned; interaction: *p* < .001 unaligned, *p* = .030 aligned). To further test whether there was any advantage of alignment during training, we compared the magnitude of the thresholds along the learning curve in Fig 3A between participants with aligned and non-aligned reference orientations, and found no difference (*F*_*(14*, *112)*_ = 1.745, *p* = .056). Likewise, there was no difference in the magnitude of thresholds for the two groups neither during testing with the 135° reference (*F*_*(4*, *32)*_ *=* 2.134, *p* = .099; data from Fig 4D), nor during testing with the 45° reference (*F*_*(4*, *32)*_ *=* 1.415, *p* = .251; data from Fig 4D). From this we conclude that the alignment of reference orientation with polar angle did not influence our results.

### 3.4 Position specificity based on immediately surrounding positions

To test whether and how specificity is related to the extent of training-induced performance enhancement, we have designed Michelson-index-based indices that separately quantity specificity and training-induced performance enhancement (see Methods). For the position specificity index, the question can be posed which peripheral positions to include into the comparison with the trained 6° position. Specifically, in section 3.2, we showed that the most peripheral test positions showed a significantly lower threshold than expected. This difference was due to an advantage at higher eccentricities and not related to learning or to an effect of cortical magnification. Therefore, including these more peripheral positions in the evaluation of position specificity could have artificially lowered specificity, and inflated generalization. To test this, we compared the effect of including the two most peripheral positions in averaged test thresholds or not. Note that this comparison was already made in Fig 3 (Section 3.1). Including the two most-peripheral test positions led indeed to a somewhat smaller averaged difference between trained and test positions (black symbols Fig 3B) compared to when only the test positions immediately surrounding the trained position were included (black symbols Fig 3C) (*t*_*(9)*_ = -2.821, *p* = .020).

Based on the above considerations, we decided for the remainder of this paper to exclude the two most-peripheral positions from the evaluation of position specificity. In addition, to verify that our main conclusions so far were not affected by the inclusion or exclusion of the two most-peripheral test positions into analysis, we re-did the main analysis in section 3.1. Hence, for the evaluation of performance at the 135° reference orientation we included only the thresholds at immediately surrounding test positions of 3° and 9° as a comparison for the thresholds in the (trained) 6° position. For consistency, we did the same with the thresholds obtained at the 45° reference orientation (grey symbols in Fig 3B and 3C). A repeated measures analysis including the data as shown in Fig 3C now yielded a significant main effect of position (*F*_*(1*, *9)*_ *=* 12.668, *p* = 0.006). Further, the re-analysis confirmed the main effect of condition (P_135_, O_45_) (*F*_*(1*, *9)*_ *=* 44.796, *p*<0.001) and the interaction between position and condition (*F*_*(1*, *9)*_ *=* 23.279, *p* = 0.001). The latter interaction was related to the larger orientation than position specificity, which was also confirmed by a t-test comparing the difference between the threshold for 135° in the trained position and neighboring positions with the difference at the trained position between the 135° and 45° reference orientation (*t*_*(9)*_ = -2.788, *p* = .021). In conclusion, using only the test positions neighboring the trained position for evaluating position specificity did not have a large effect on the data and did not affect our main conclusions of limited but significant position and orientation specificity. Nevertheless, for the quantification of position specificity (PS) in the following sections, we compared the trained position with only the two neighboring test positions, as this is conceptually the fairest comparison given the eccentricity effect described in Section 3.2.

Note that to relate orientation specificity to the extent of training, we used the Michelson-index-based orientation specificity index OS, which compares performance at the 6° position between the trained 135° reference and the untrained 45° reference (see Methods). Thus, for OS, there is no issue related to which test positions to include into the calculation of specificity.

### 3.5 Position specificity is related to the extent of late learning

The position specificity effects reported in Figs 2–4 were small compared to overall learning. For more insight into the relative size of the training and position specificity effects, we calculated and compared training and position specificity indices. To make a comparison between an index of training extent and position specificity, it is important that these indices are based on non-overlapping datasets. Hence, we used the PS position specificity index in the present section, which gives a normalized threshold increase in test compared to trained position, based on data that are different from those used to quantify the training index T (for details, see Methods). A paired sample t-test showed that the training index (T = 0.40) was larger than the position specificity index (PS = 0.19) *(t*_*(9)*_ = 4.740, *p* = 0.001). When including all available data (extra thresholds 61–80 in P2-4 and refresher thresholds in P8-10), T was 0.43.

**Fig 5:**
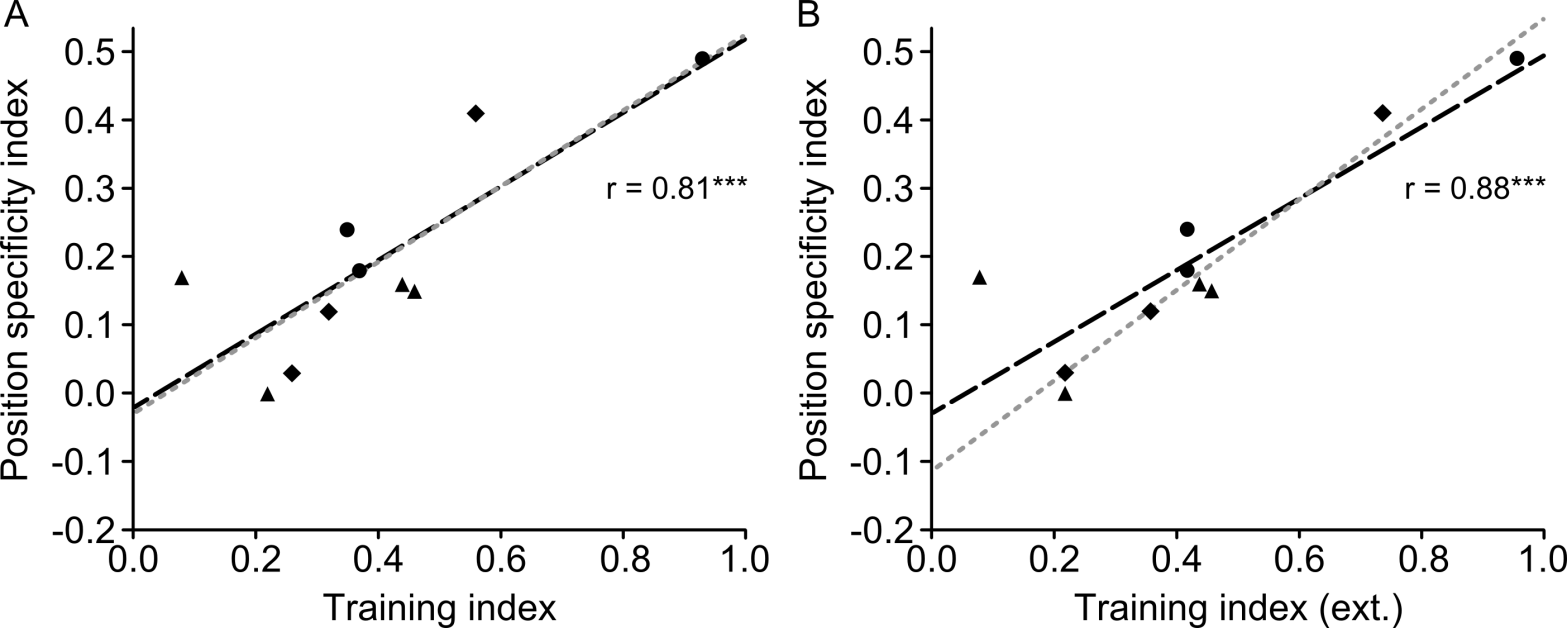
Scatterplot of position specificity and training indices. ***A***, Position specificity correlates positively with the training index. ***B***, The correlation between position specificity and training index was even stronger when using the complete learning data (including extra thresholds and refresher training) for calculation of the training index. The black dashed line results from an ordinary least square regression (r also obtained from that analysis), whereas the grey dotted line was fitted with betas obtained by using robust regression. Different symbols represent different training schemes: triangle = 15 sessions with 4 staircases, circle = 10 sessions with 8staircases, diamond = 15 sessions with 4 staircases plus 3 refresher sessions. * *p* < .050, ** *p* < .010, *** *p* < .005.

Using the data from the first 60 thresholds in all participants, we tested whether the training index was related to the position specificity index, and found a positive correlation (*r* = .81, R^2^ = 0.66, *p* = .004). This indicates that the more participants improved during the training phase, the greater the position specificity effect was (Fig 5A). The training index significantly predicted position specificity (β = 0.54, *t*_*(9)*_ = 3.944, *p* = .004) and also explained a significant proportion of variance in position specificity (R^2^ = 0.66, *F*_*(1*,*9)*_ = 15.6, *p* = .004). Robust regression yielded a slightly higher beta weight (β = 0.55, *t*_*(9)*_ = 3.683, *p* = .006) indicating that outliers did not strongly drive the OLS regression results. This pattern of results was confirmed when including all available training data in all participants (i.e. estimating end of learning on thresholds 67–80 in P2-4 and end of refreshing session in P8-10) (β = 0.52, *t*_*(9)*_ = 5.216, *p* = .001, *r* = .88, R^2^ = 0.69, *F*_*(1*,*9)*_ = 17.7, *p* = .003) (Fig 5B).

**Fig 6:**
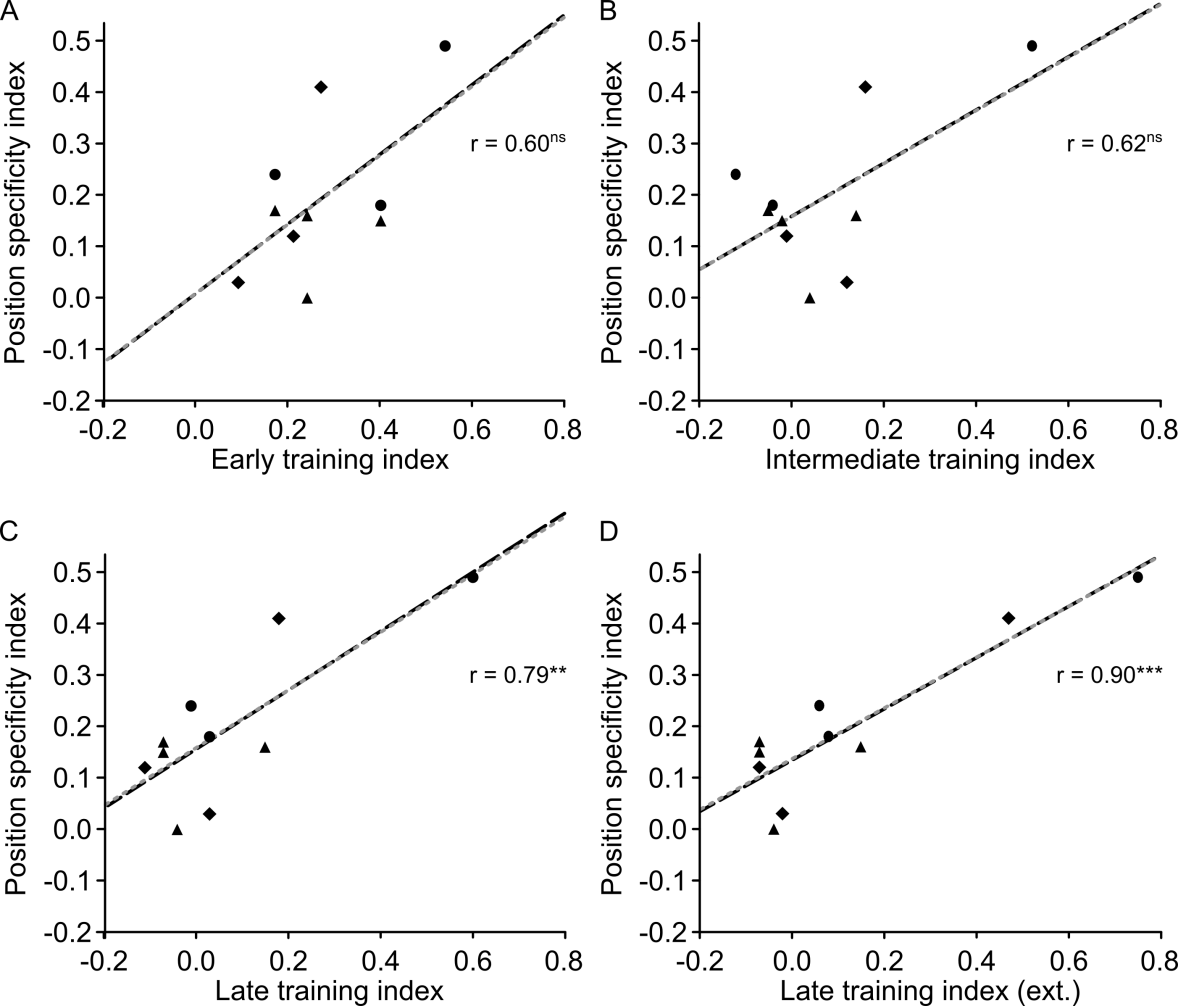
Scatterplot of position specificity and early (***A***), intermediate (***B***), and late training indices either based on 60 thresholds learning data (***C***) or on additional asymptotic data for participants P2-4 and P8-10 (***D***). The black dashed line results from an ordinary least square regression (r also obtained form that analysis), whereas the grey dotted line was fitted with betas obtained by using robust regression. Different symbols represent different training schemes: triangle = 15 sessions with 4 staircases, circle = 10 sessions with 8staircases, diamond = 15 sessions with 4 staircases plus 3 refresher sessions. * *p* < .050, ** *p* < .010, *** *p* < .005.

To further explore the relationship between the training index and the position specificity index, we correlated the position specificity index with the training indices from session 1–5, session 6–10, and session 11–15, referred to as early, intermediate and late training index, respectively. While the early and intermediate training indices (Fig 6A and 6B) did not significantly correlate with the position specificity index (early: *r* = .60, *R*^*2*^ = 0.36, *p* = .069; intermediate: *r* = .62, *R*^*2*^ = 0.38, *p* = .058), there was a highly significant correlation for the late training index (Fig 6C) (late: *r* = .79, *R*^*2*^ = 0.62, *p* = .007). When basing the end of late learning in participants P2-4 and P8-10 on the additional asymptotic data (Fig 6D), the correlation between position specificity index and late training index was even stronger (*r* = .90, *R*^*2*^ = 0.81, *p* < .001). This indicates that the magnitude of late (asymptotic) learning contributed to the degree of position specificity.

A model combining early, intermediate and late learning as predictors was precluded by multicollinearity (intermediate and late learning: *r* = 0.94). Therefore, separate models for each of the three predictors were calculated (using the 60-threshold training data). Early and intermediate learning were not a significant factor for predicting position specificity (early: *β* = 0.67, *t*_(9)_ = 2.103, *p* = .069; intermediate: *β* = 0.52, *t*_(9)_ = 2.206, *p* = .058), whereas late learning significantly affected position specificity (late: *β* = 0.57, *t*_(9)_ = 3.584, *p* = .007).

Late learning explained a significant proportion of variance in position specificity (*R*^2^ = 0.62, *F*_(1,9)_ = 12.8, *p* = .007) whereas early and intermediate indices did not significantly explain variance (early: *R*^*2*^ = 0.36, *F*_(1,9)_ = 4.42, *p* = .069; intermediate: *R*^*2*^ = 0.38, *F*_(1,9)_ = 4.87, *p* = .058). Since in intermediate and late training indices, there was one data point that qualified as an outlier according to a 1.5 IQR criterion (P2), we used a robust regression to see how that data point influenced the estimates. Beta weights from robust regression were almost identical (early: *β* = 0.67, *t*_(9)_ = 1.928, *p* = .090; intermediate: *β* = 0.52, *t*_(9)_ = 2.060, *p* = .073; late: *β* = 0.56, *t*_(9)_ = 3.249, *p* = .012), indicating a negligible contribution of outliers to the OLS regression analysis. When including the training data from extra thresholds in six participants (P2-4 and P8-10) into calculations of the late training index, the overall picture remained the same (*β* = 0.50, *t*_(9)_ = 5.821, *p* < .001; *R*^2^ = 0.81, *F*_(1, 9)_ = 33.9, *p* < .001).

To assess the difference between correlations of training to specificity indices, we performed Lee and Preacher's test for differences between dependent correlations [72]. As we had the hypothesis that training to position specificity correlations would become larger for later phases in learning, we used a one-sided test. No evidence was found in support of a larger correlation for intermediate compared to early learning (*p* = .470). However, the correlation for late learning showed a slight non-significant trend to be smaller than for early learning (*p* = .177), and a significant trend to be smaller than for intermediate learning (*p* = .016). In addition, the correlation for late-extra learning was significantly lower than for early learning (*p* = .034) and for intermediate learning (p = .001). Note that the late-extra condition refers to the inclusion of extra thresholds in six participants (P2-4 and P8-10) into calculations of the late training index. Overall, we suggest that this pattern of differences among correlations supports the relationship we propose between the extent of asymptotic learning and specificity (see also Table 2).

**Table 2:** Comparison of correlations between training and position specificity indices for different parts of the learning curve.

correlations of training and specificity indices compared for:	one-tailed *p* position specificity
Early vs. Intermediate	0.470
Early vs. Late	0.177
Intermediate vs. Late	0.016
Early vs. Late-extra	0.034
Intermediate vs. Late-extra	0.001

In the late-extra condition, the training data from extra thresholds in six participants (P2-4 and P8-10) were included into calculations of the late training index.

It is possible that random factors lead to larger variability in estimating early and intermediate learning compared to later learning, which might be responsible for the lack of significant correlations between progress in early training and position specificity. We therefore checked the variability in all training indices. The variance in the overall training indices was 0.05, and the variances for the training indices in the tertiles of learning were 0.02 for early training, 0.03 for intermediate learning, and 0.04 for late learning. The variance in late learning when using all available data increased to 0.08 (i.e., including refresher thresholds in P8-10 and thresholds 61–80 in P2-4). The levels of variance for the training indices of the three tertiles of the learning curve did not differ significantly (Levene’s test for homogeneity of variances: *F* = 0.303, *p* = .741), and if anything, the early learning tended to showed smaller rather than larger variance. Therefore, this control analysis supports the proposed positive relationship between the extent of late asymptotic learning and magnitude of position specificity.

### 3.6 Orientation specificity is related to the extent of late learning

As done for position specificity, we calculated and compared training and orientation specificity indices. A paired sample t-test showed that the training index (T = 0.40)–based on the first 60 thresholds as shown in Fig 3 —was larger than the orientation specificity index (OS = 0.26) (*t*_*(9)*_ = 2.755, *p* = 0.022).

**Fig 7:**
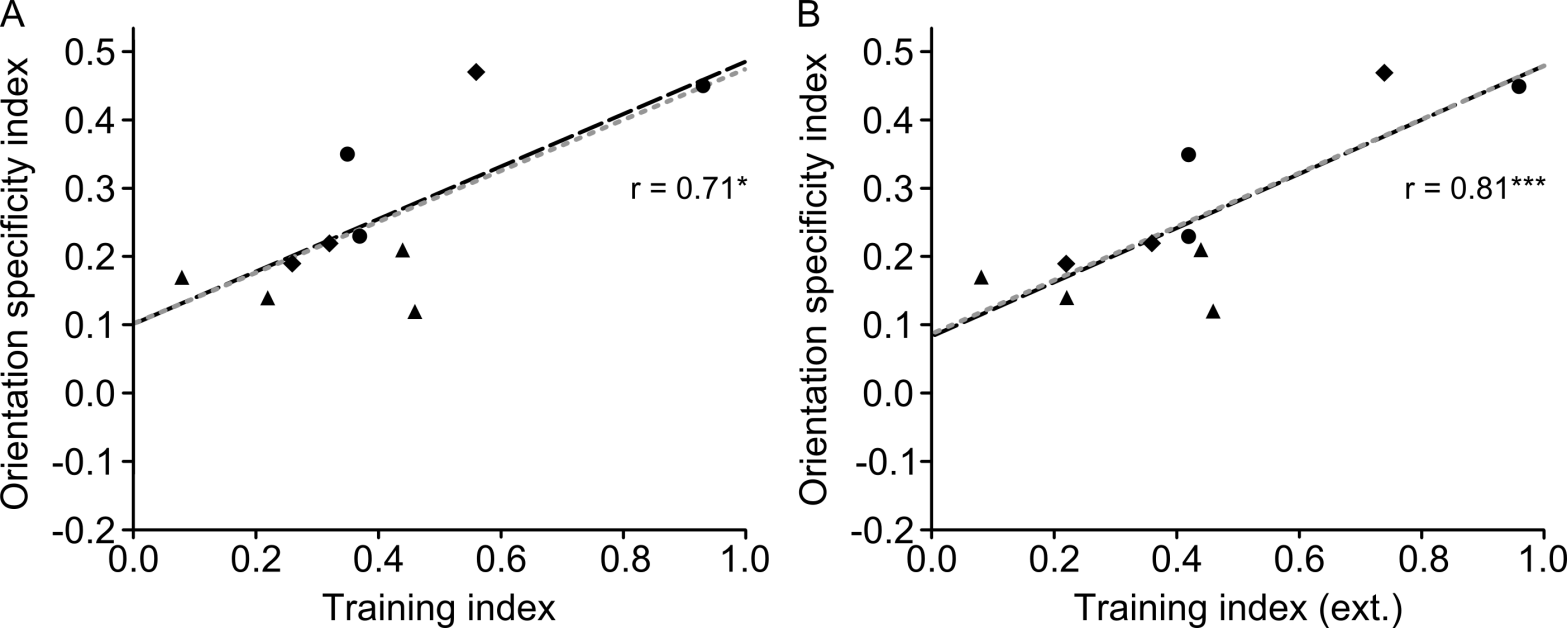
Scatterplot of orientation specificity and training indices. ***A***, Orientation specificity correlated positively with the training index. ***B***, The correlation between orientation specificity and training index was even stronger when using the complete training data (including extra thresholds and refresher training) for calculation of the training index. The black dashed line results from an ordinary least square regression, whereas the grey dotted line was fitted with betas obtained by using robust regression. Different symbols represent different training schemes: triangle = 15 sessions with 4 staircases, circle = 10 sessions with 8staircases, diamond = 15 sessions with 4 staircases plus 3 refresher sessions. * *p* < .050, ** *p* < .010, *** *p* < .005.

Using the data from the first 60 thresholds in all participants, we then tested whether the training index was related to the orientation specificity index in a similar way as the position specificity index, and found a positive correlation (r = .71, R^2^ = 0.51, *p* = .021). Similar to the position specificity index, the more participants improved during the training phase, the greater the orientation specificity effect was (Fig 7A). The training index significantly predicted orientation specificity (β = 0.39, *t*_*(9)*_ = 2.863, *p* = .021) and also explained a significant proportion of variance in orientation specificity (R^2^ = 0.51, *F*_*(1*,*8)*_ = 8.20, *p* = .021). Robust regression yielded a slightly lower beta weight (β = 0.37, *t*_*(9)*_ = 2.498, *p* = .037) indicating that outliers did not drive the OLS regression results strongly. This pattern of results was confirmed when including all available training data in all participants (i.e. estimating end of learning on thresholds 67–80 in P2-4 and end of refreshing session in P8-10) (β = 0.40, *t*_*(9)*_ = 3.967, *p* = .004, r = .81 R^2^ = 0.66, *F*_*(1*,*9)*_ = 15.70, *p* = .004) (Fig 7B).

**Fig 8:**
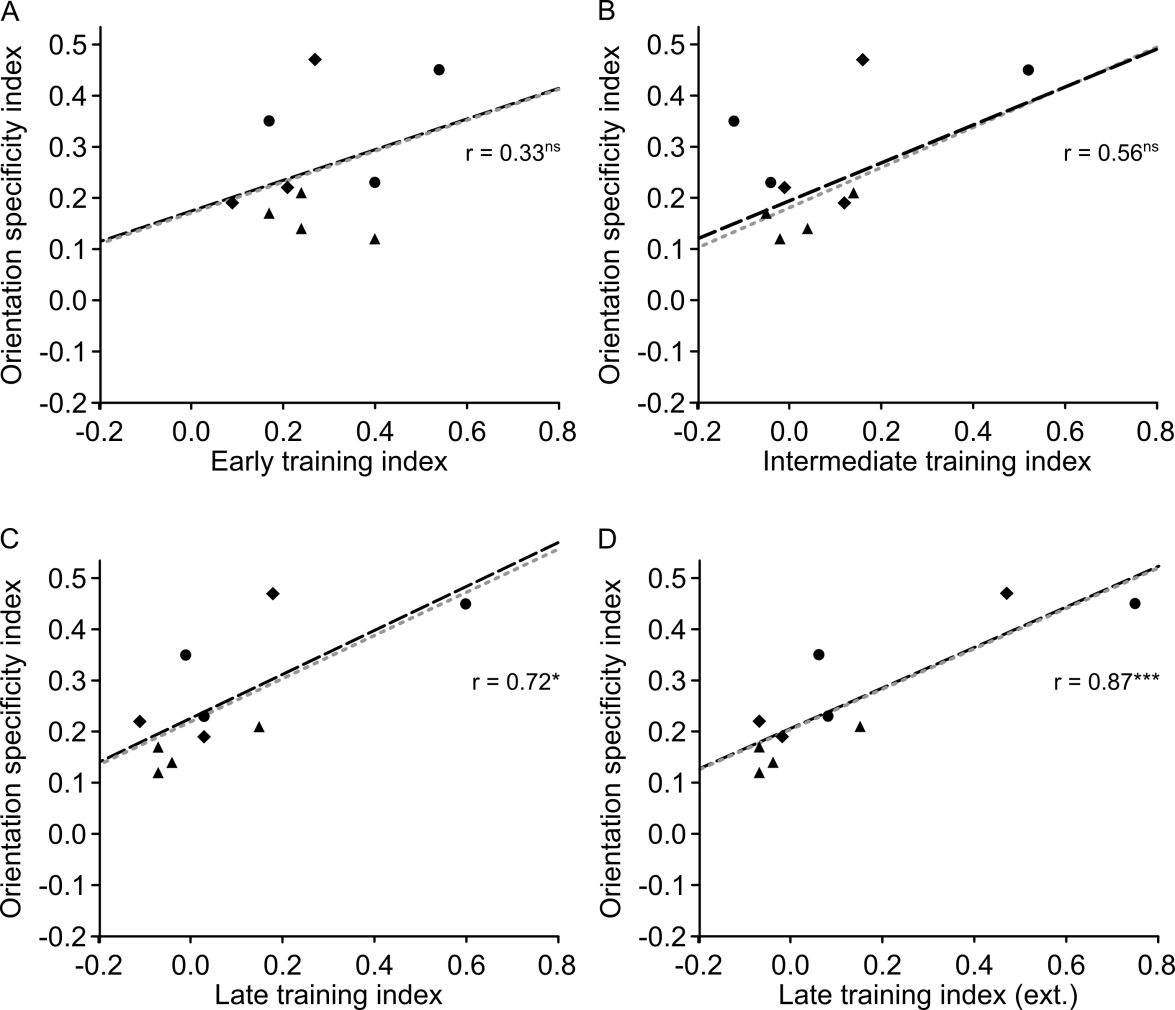
Scatterplot of orientation specificity and early (***A***), intermediate (***B***), and late training indices either based on 60 thresholds learning data (***C***) or on the same data including additional asymptotic data for participants P2-4 and P8-10 (***D***). The black dashed line results from an ordinary least square regression, whereas the grey dotted line was fitted with betas obtained by using robust regression. Different symbols represent different training schemes: triangle = 15 sessions with 4 staircases, circle = 10 sessions with 8staircases, diamond = 15 sessions with 4 staircases plus 3 refresher sessions. * *p* < .050, ** *p* < .010, *** *p* < .005.

To further explore the relationship between training index and orientation specificity index, we also correlated the orientation specificity index with the early, intermediate and late training indices. While the early (Fig 8A) and intermediate (Fig 8B) training indices did not correlate with the orientation specificity index significantly (early: *r* = .33, *R*^*2*^ = 0.11, *p* = .358; intermediate: *r* = .56, *R*^*2*^ = 0.31, *p* = .092), there was a significant correlation for the late training index (Fig 8C), which was based on a comparison between thresholds 41–44 and thresholds 57–60 (*r* = .72, *R*^*2*^ = 0.52, *p* = .019). When basing the end of late learning in participants P2-4 and P8-10 on the additional asymptotic data (Fig 8D), the correlation between orientation specificity index and late training index was even stronger (*r* = .87, *R*^*2*^ = 0.76, *p* = .001). This indicates that the magnitude of learning in the late asymptotic phase contributed to the degree of orientation specificity. Having multicollinearity between intermediate and late learning, only separate models for each of the three predictors were calculated (using the 60-threshold training data). Early and intermediate learning were not a significant factor for predicting orientation specificity (early: *β* = 0.30, *t*_(9)_ = 0.975, *p* = .358; intermediate: *β* = 0.39, *t*_(9)_ = 1.912, *p* = .092), whereas late learning significantly affected orientation specificity (*β* = 0.43, *t*_(9)_ = 2.945, *p* = .019). Late learning explained a significant proportion of variance in orientation specificity (*R*^2^ = 0.52, *F*_(1, 9)_ = 8.68, *p* = .019). Beta weights from robust regression were almost identical (early: *β* = 0.30, *t*_(9)_ = 0.903, *p* = .393; intermediate: *β* = 0.40, *t*_(9)_ = 1.823, *p* = .106; late: *β* = 0.42, *t*_(9)_ = 2.684, *p* = .028), indicating a negligible contribution of outliers to the OLS regression analysis. When including the training data from extra thresholds in six participants (P2-4 and P8-10) into calculations of the late training index, the overall picture remained the same (*β* = 0.39, *t*_(9)_ = 4.972, *p* = .001; *R*^2^ = 0.76, *F*_(1, 9)_ = 24.70, *p* = .001).

Note that for the correlation analysis that includes the late-extra data (6 participants, see Table 3), it should be acknowledged that the further boost in the positive correlation between specificity and training index incurs a limit in interpretability. This is due to differences in the numbers of sessions of late-extra training among these 6 participants, and between these 6 participants and the other participants in which the late-extra data were not available (and where hence the training index for the late training condition was entered into the correlations). This means that the progress in the late-extra condition over participants was determined both by whichever mechanisms played a role during asymptotic learning and by the duration of the asymptotic learning. This should be taken into account in interpreting the correlations involving the late-extra condition. Nevertheless, the strengthened correlation between specificity and the training-indices including late-extra data affirm the importance of asymptotic learning in inducing specificity.

**Table 3:** Correlations between training indices for six participants with late-extra data.

	*T_early*	*T_middle*	*T_late*	*T_ext_late*
T_early	1			
T_middle	0.64	1		
T_late	0.77	0.94	1	
T_ext_late	0.75	0.86	0.95	1

In the late-extra condition, the training data from extra thresholds in six participants (P2-4 and P8-10) were included into calculations of the late training index. Consequently it becomes possible to correlate this extra index with the remaining training indices for this subgroup of participants only.

To assess the difference between correlations of training indices with orientation specificity for different parts of the learning curve, we performed Lee and Preacher's test for differences between dependent correlations [72]. Because we had a specific hypothesis about the direction of the differences, we used a one-tailed statistic. Table 4 shows no support for the hypothesis that the training index versus orientation specificity correlation was higher for the intermediate compared to the early part of learning (*p* = .277). However, the training index versus orientation specificity correlations for the late-extra and the late training phases tended to be significantly lower compared to correlations for earlier phases of the training (for an overview see Table 4). This supports the relationship we propose between the extent of asymptotic learning and specificity (see also section 3.5).

**Table 4:** Comparison of correlations between training and orientation specificity indices for different parts of the learning curve.

correlations of training and specificity indices compared for:	one-tailed *p* orientation specificity
Early vs. Intermediate	0.227
Early vs. Late	0.057
Intermediate vs. Late	0.040
Early vs. Late-extra	0.003
Intermediate vs. Late-extra	0.001

In the late-extra condition, the training data from extra thresholds in six participants (P2-4 and P8-10) were included into calculations of the late training index.

Moreover, the variability in the training indices used for the analyses were not significantly different (Levene’s test for homogeneity of variances: all *p* > .077), and thus did not explain differences in correlations. This pattern of differences between correlations further supports the relationship between the extent of asymptotic learning and specificity. Note that the training indices included in Fig 2 (for complete learning curve and the three tertiles) per participant, can be combined with the specificity indices in Table 5 to yield the training index to specificity index correlations studied in sections 3.5 and 3.6. It is also noteworthy that the orientation specificity and position specificity indices were highly correlated (*r* = .90, *R*^*2*^ = 0.81, *p* < .001; see Table 5).

**Table 5:** Orientation and specificity indices for individual participants.

	PS all	PS surround	OS
**pp1**	0.16	0.16	0.21
**pp2**	0.40	0.49	0.45
**pp3**	0.14	0.18	0.23
**pp4**	0.19	0.24	0.35
**pp5**	0.14	0.17	0.17
**pp6**	0.09	0.15	0.12
**pp7**	0.04	0.00	0.14
**pp8**	0.11	0.12	0.22
**pp9**	0.02	0.03	0.19
**pp10**	0.32	0.41	0.47

PS all refers to the Michelson-based position specificity index taking into account all surrounding test positions; PS surround refers to the Michelson-based position specificity index computed on just the two immediately surrounding test positions. OS refers to the Michelson-based orientation specificity index. For the computation of correlations between position specificity and training indices, we only used the PS surround indices.

The above arguments all support the idea that specificity (both for orientation and position) is correlated stronger with late asymptotic learning than with early learning. This in turn suggests that the learning mechanisms that drive performance increments are different during earlier compared to late stages of learning. Specifically, we expected that the correlation in training index between the late tertile of learning and early tertile of learning (r = 0.63) would be significantly lower than between the late tertile of learning and the middle tertile of learning (r = 0.94). We tested this using Lee and Preacher’s test for differences between dependent correlations and found this to be the case (Z = -2.17, p = 0.015, one-tailed). We also found that the middle-to-late correlation (r = 0.94) was significantly larger than the middle-to-early correlation (r = 0.55) (Z = -2.73, p = 0.003, one-tailed). These confirm that especially in the early part of the learning training-induced performance enhancements are driven by mechanisms that are rather different from those playing a role in the late part, with the middle part probably representing a transition from one to the other type of mechanisms. We also computed the correlations in training index between the late-extra part of the learning curve and preceding parts, and found trends compatible with this view, with statistical tests narrowly missing significance due to the fact that only 6 participants had data in the late-extra condition (see Table 3).

### 3.7 Details of training scheme or differences in experimental context among participants do not drive correlations between training effects and specificity

It is possible that differences in training scheme or experimental context would cause individual differences in either training indices or specificity indices, which could drive the correlation between both. Therefore, we first checked for all *training indices* whether their mean differed among different training schemes (15x4, 10x8, 15x4-Refresh) or experimental context (none, study A, study B, or study A & B), using a one-way ANOVA. The analysis showed that none of the training indices differed between groups for training scheme (all *F*-values < 1.501 and all *p*-values > .287) or experimental context (all *F*-values < 1.442 and all *p*-values > .321), showing that between-group variance was not significantly bigger than within-group variance. These results thus indicate that progress in training (over the learning curve as a whole, or in first, second and third tertile) is unrelated to training scheme or experimental context.

Second, we also performed the same analyses on the orientation and position specificity indices and one-way ANOVAs similarly showed no difference between groups for training scheme (all *F*-values < 2.939 and all *p*-values > .118), or experimental context (all *F*-values < 2.003 and all *p*-values > .215). Variation in specificity indices among participants is thus not due to differences in training scheme or experimental context. Since both the (position/orientation) specificity indices and the training indices were not influenced by training scheme or experimental context, it seems reasonable to conclude that specificity shows a genuine correlation with training index.

Moreover, we did an additional analysis to test the effects of amount of training in previous studies (related to experimental context) and differences in amount of training in the present study (related to training scheme). As seven of our participants had participated in other perceptual learning studies (with training in one or sometimes three quadrants other than the one used for within-quadrant position specificity testing), we were interested whether the number of trained orientations, quadrants and trials at which they had been previously trained (*see* Table 6) influenced the observed specificity effects or the level of the asymptote defined as the last 20 thresholds obtained. Furthermore, the number of trials spent in the present study (*see* Table 6) differed among participants due to the extra thresholds and refresher thresholds, which was a potential confounder. We therefore correlated the position specificity index, the orientation specificity index, and asymptotic performance level with these four factors. No significant correlations were found (Table 7). Thus, neither the numbers of trials spent in previous studies (in quadrant(s) other than the one used for the present study), nor the variation among participants in numbers of trials spent in the present study seemed to be determining factors in explaining position and orientation specificity within the quadrant used in the present study.

**Table 6:** Amount of previous training.

	previous studies			present study
	nr. trained orientations	nr. trained quadrants	nr. of trials	nr. of trials for training & refresh
P1	3	2	15669	5352
P2	0	0	0	7193
P3	0	0	0	6711
P4	0	0	0	7146
P5	1	4	17308	5127
P6	3	2	15748	5356
P7	1	4	16013	5302
P8	3	2	15826	8068
P9	3	4	36206	8690
P10	3	4	36847	8711

Number of trained orientations, trained quadrants, and trials for any previous experiments and number of trials performed during training and refresh sessions for the present experiment. Note that participants P2-4 performed 80 thresholds during training whereas all other participants completed only 60 thresholds.

**Table 7:** Effect of previous training.

		previous studies			present study
		N trained reference orientations	N trained quadrants	N trials	Total N trials for training & refresh
position specificity index	r	0.34	0.35	0.20	0.18
	R^2^	0.12	0.13	0.04	0.03
	p	0.330	0.315	0.581	0.621
orientation specificity index	r	0.31	0.30	0.08	0.48
	R^2^	0.09	0.09	0.01	0.23
	p	0.387	0.399	0.836	0.158
last 20 thresholds (asymptote)	r	0.23	0.51	0.31	0.41
	R^2^	0.05	0.26	0.10	0.17
	p	0.514	0.131	0.376	0.235

Correlation, explained variance and *p*-values for different correlations. Upper part shows correlation of position specificity index with number of trained orientations, trained quadrants, and trials of previous studies and number of trials during training for the present study. Middle part shows correlations of orientation specificity index with the same factors. Lower part shows correlations of last 20 thresholds with the same factors mentioned above.

## 4 Discussion

The study presents two main findings. First, we show that extensive orientation discrimination training at a single location in a visual field quadrant yields partial but significant position and orientation specificity. Despite significant specificity, we found more generalization in the present study within a quadrant than was observed across quadrants or hemifields in another recent study from our group [68]. The greater within-quadrant generalization may reflect contributions of horizontal connectivity within early visual areas [73]. Second, position and orientation specificity indices correlated strongly and positively with the total performance increment achieved during the training. Moreover, when comparing the relationship between specificity and training indices in the first, middle and last tertile of the learning curves, we found that the more improvement was made during later training phases, the larger the specificity of learning was. This relationship was not present during the first and middle tertile of the learning curve. This is to our knowledge the first evidence quantitatively linking training effects in the later phases of learning with specificity, in agreement with proposals in older studies [1, 4, 50] that asymptotic learning is necessary to achieve position (and stimulus) specific perceptual skill. In line with this, the correlation in training progress between early and late parts of the learning curves was substantially lower than that between early and intermediate parts. This supports the idea that the mechanisms that underlie training-indices performance enhancements in early and late parts of the learning curve are different, in accordance with our proposal that mechanisms of specificity/generalization are also different after early and late learning. Additionally, we had designed our study to test whether the spatial distribution of transfer around a trained location would be influenced by the cortical magnification of central vision in retinotopic visual areas, but did not find support for this idea.

### 4.1 Specificity is more related to late learning, than to early and intermediate learning

Our results are relevant for studies that have been appearing over the last decade suggesting almost complete generalization of learning in a ‘double training’ paradigm [21, 59]. In these studies, a brief period of training in an irrelevant task at a first location, followed by training in a second task in another location, led to generalization of the skill acquired in the second task towards the first location. Based on these findings, it was suggested that the idea of specificity as a core property of perceptual skill learning may be altogether incorrect. They suggested instead that visual perceptual learning may be rule-based, and hence generalization would reflect processes taking place independent of low-level properties of low-level visual areas. In this view, there should be full generalization, or generalization should be easily triggered in the context of specific training paradigms [21, 59]. In the present study, however, even for neighboring positions within a single quadrant, we found partial but significant position and orientation specificity. The position and orientation specificity tests in the present study showed threshold increases between trained and test positions matching a threshold increase from session 15 to session 8 (position specificity) or from 15 to 4 (orientation specificity), which is approximately where Xiao et al. [21] and J.-Y. Zhang et al. [59] ended their training. We suggest, in line with other studies, that there is a strong tendency for generalization in the first parts of the learning curve [17]. Hence, Xiao et al.’s [21] findings can be seen as revealing conditions in which learning transfer can be facilitated in the early phase of learning. It is possible that part of the mechanisms leading to generalization during early learning is rule-based. However, the mechanisms underlying the amount of transfer (i.e., the amount of specificity) in the present study were unrelated to early learning, as indicated by a lack of a correlation in our study between position/orientation specificity and performance improvements in early learning. In line with this, a recent study [68] from our group showed that brief pretesting at the beginning of learning, followed by extensive orientation discrimination training in another location, led to a small facilitation of transfer towards the pretested location, which however excluded the performance gains that had been made during asymptotic learning. Our findings thus indicate that mechanisms and conditions leading to specificity/generalization of learning are different for early and asymptotic learning, in line with our correlation data that suggests also differences in the learning mechanisms driving performance increments in earlier and later parts of the learning curve.

These outcomes are not predicted by the theoretical framework of double training studies, but gives the first quantitative support for the prominent idea in older literature relating specificity to asymptotic learning (Fahle, 1997; Karni & Bertini, 1997; Karni et al., 1995; Karni & Sagi, 1991; Schoups et al., 1995; Schwartz, Maquet, & Frith, 2002). The relationship between specificity and the extent of asymptotic learning helps to evaluate possible neural mechanisms underlying specificity. The relationship we documented fits well with training-induced orientation tuning changes in V1 (Raiguel, Vogels, Mysore, & Orban, 2006; Schoups, Vogels, Qian, & Orban, 2001) (Schoups et al., 2001 and V4 (Raiguel et al., 200x), although these plastic effects were reported only after training considerably longer than in the present study. Conversely, studies that consider specificity in the domain of perceptual skill learning as conditional or rule-based typically used restricted training durations, thereby likely limiting contributions from the lowest levels of the visual system. Thus, documenting a quantitative relationship between the magnitude of asymptotic learning and specificity helps to understand mechanisms of skill learning and its properties. However, the relationship between asymptotic learning and specificity cannot distinguish between the lowest-level and reverse hierarchy theories, because both predict the same relationship.

### 4.2 The within-quadrant spatial gradient in specificity/transfer appears unrelated to retinotopic mechanisms

To test a potential contribution of low-level visual cortex to the spatial gradient of generalization/specificity, we have tested this generalization gradient from a trained peripheral location towards foveal vision and towards peripheral vision. We had reasoned that generalization should be influenced by cortical magnification if it were generated by plasticity within early visual cortex, with generalization over a smaller distance towards central vision and over a larger distance towards the periphery. No such asymmetry was expected should generalization be generated at high levels of the visual system not characterized by cortical magnification or retinotopy (see Introduction).

We collected data during generalization testing that at first sight argued in favour of a retinotopic mechanism of generalization. Based on control analyses and a control experiment in naïve participants, we had to conclude that the observed data did not reveal a mechanism of generalization, but rather reflected a sensory-based interaction between the Gabor stimulus we used and eccentricity. This is because, surprisingly, we observed better orientation discrimination performance with increasing eccentricity even in naïve participants. This contrasts with the more common finding that visual performance declines with eccentricity, also referred to as the ‘eccentricity effect’ (e.g., [74, 75]). Eccentricity effects in orientation discrimination however have been documented in only a few studies [75–78]. Interestingly, among those studies, Vandenbussche et al. [78] reported that orientation discrimination of a single line at oblique standard orientations did not change at all with eccentricity, in contrast to principal orientations. The cited studies investigating orientation discrimination that did find an eccentricity effect [75–77], used a vertical reference orientation. The lack of the expected eccentricity effect in our data hence is to some extent in line with Vandenbussche et al. [78]. Nevertheless, the increase in performance with eccentricity in our data (in 20 participants) is remarkable, and asks for new parametric investigations of the effect of eccentricity on naïve and skilled perception of gratings or Gabor stimuli using a range of spatial frequencies and contrasts.

Hence, we could not resolve the question whether retinotopic or reverse hierarchy read-out mechanisms contribute to the specificity that emerges during asymptotic learning. To do so would require finding a stimulus that is equally well discriminated at a training location and neighboring equipolar test locations. In addition, it is possible that the present training durations were too limited to reveal the full impact of low-level tuning changes and of cortical magnification effects on the spatial generalization gradient. Notably, low-level tuning changes in V1 and V4 have been reported after much longer training durations (of several months, see [8, 12]) than those applied in the present study. Hence, future studies of specificity in perceptual skill learning may benefit from comparing the properties of generalization between shorter periods of training (e.g., seven sessions) and much longer periods of training (e.g., 30 or more sessions). It is possible that in experimental designs similar to ours, more extended training will reveal a pattern of specificity compatible with contributions of cortical magnification in retinotopic areas.

### 4.3 Other factors affecting specificity

With respect to our findings of (partial) specificity and its relationship with the extent of performance enhancement during asymptotic learning, we have done a number of control analyses to exclude alternative explanations. One interesting finding from these analyses is that *differences in experimental context* among participants (who underwent varying amounts of training in other quadrants for other experiments not included in the present paper) *did not affect estimates of within-quadrant specificity/transfer*, *or correlations between specificity and amounts of training*. Although the different history of our participants could be perceived as a downside, this finding is interesting as it indicates there is limited transfer of skill across quadrants, at least when the skill is sufficiently trained. This is in line with previous work from our group showing that different quadrants subjected to different experimental treatments during extensive learning yield very different thresholds [66]. Different experimental manipulations to influence learning can therefore be applied in different quadrants in a fairly independent manner. This is also in line with another recent study [68] that demonstrated almost no transfer after extensive training among relatively distant locations in the visual field. Other studies that used extensive training in different quadrants also have reported relatively robust specificity [4, 7, 8]. Hence, we suggest that the irrelevance of training history in quadrants other than the one considered in the present study for the assessment of specificity is consistent with existing literature.

Another finding coming out of our control analyses was that the *variation in position specificity and orientation specificity among participants in our study was not correlated significantly with the variation in the number of trials during training among participants*. This seems to conflict with findings of Jeter et al. [17], who reported that more training trials lead to more specificity. To interpret our finding, it is useful to compare the numbers of trials used in our study with those in Jeter et al. [17]. In our experiment, participants were trained over a period of ten to fifteen sessions and completed a total of 60–80 staircases which amounted to total training trial numbers ranging between 5127 and 8711 when including the extra trials in P2-4 and the refresher trials in P8-10 (see Table 2). Jeter et al. [17] report that shorter training (i.e., up to 2496 trials) resulted in transferable performance levels, whereas extensive training (i.e., 4992 or 7488) resulted in partial specificity. Given the overall number of trials (mean number of trials 6766) it is therefore fully in line with Jeter et al.’s results that we see specificity in our data. It is possible that the range in total trial numbers among participants was not sufficient to induce a sufficiently large variation in position specificity to produce a correlation between total trial numbers and position specificity.

Another factor that can play a role in influencing specificity is the number of trials within a session, which, when sufficiently large could lead to adaptation effects that could enhance the specificity of learning [57]. Despite that fact that some participants performed more trials per session than other participants (see Table 1), these *variations in numbers of trials per sessions did not lead to differences in specificity among participants*, *which might be due to the considerable total length of training both in terms of sessions and trials*. In our study the number of trials per session was relatively low for the seven participants doing 15 training sessions (mean of 354 trials/session) and higher for three participants (P2-4) doing ten training sessions (mean of 702 trials/session). Situated in the entire group of ten participants, the specificity index was largest in P2, sixth largest in P3, and third largest in P4. Thus, the specificity indices for P3 and P4 fell within the range of the other participants, and outlier analysis showed that the index for P2 fell within the normal range. It therefore seems unlikely that there were strong additional adaptation effects in P2-4 that would have led to greater position specificity in those subjects, and hence in our hands, sessions with roughly 350 or roughly 700 trials per session can both lead to specificity. This contrasts with Aberg et al. [15], who observed generalization in a chevron task with 400 trials per session and specificity with 800 trials per session with the overall amount of training being identical.

The fact that a reduction in the number of trials per session from ~700–800 trials to ~350–400 trials led to the disappearance of specificity in Aberg et al.’s (14) study, whereas in ours a similar reduction did not eliminate specificity might be understood in the context of differences in the total number of trials and amount of asymptotic learning. Aberg et al. [15] only used 1600 trials for the total length of training, whereas we used 5127–8711 trials. Hence, our finding of partial specificity to the trained position, even with rather low trial numbers per session for seven participants, might be explained by the more extensive total amount of training in our study, which continued until participants reached an asymptotic performance level.

Recently, Hung and Seitz [56] showed in an orientation discrimination task that there was greater transfer to another location for training with multiple short staircases in contrast to a single, long staircase [56]. Our training procedure resembled that of Hung and Seitz (26) in the sense that the start level of each staircase in a given session was set to the average threshold level of the previous session. Hence, the specificity in our data and theirs could be related to the presentation of many trials at threshold. It could be asked whether the lack of easy orientation differences at the start of individual staircases was the sole reason for the (partial) specificity observed in Hung and Seitz’ (26) study and in ours. With respect to that question, it is relevant that in Hung and Seitz (26), final thresholds prior to generalization testing appeared to be larger in the multiple compared to the single staircase condition, which favored generalization to the other location given that performance in the test location following training in the multiple versus single staircases conditions was about the same. Moreover, the intermixing of high with threshold-level orientation differences may have counteracted reaching the best performance level, thus preventing the task from increasing the challenge, which in turn may have favored generalization. Thus we suggest that it is not the lack of easy orientation differences at the start of individual staircases in our study that induced specificity. We suggest that any manipulation that prevents participants from reaching the stage of cumulative asymptotic learning at threshold will limit specificity and favor generalization. Compared to the 6 sessions of training performed by participants in Hung and Seitz (26), participants in our study performed 10–19 sessions, which may have promoted asymptotic learning and specificity.

In the context of the reviewed studies, our finding that the number of trials does not play a role in predicting differences in specificity among participants suggests that the lowest numbers of trials administered (in total, and per session) are high enough to induce a ceiling level of plasticity. As a consequence, trial numbers in themselves are not a determinant of the magnitude of learning or specificity. Instead, differences in learning and specificity among participants may reflect differences in the capacity for plastic change in their visual systems, rather than differences in induced plasticity due to differences in numbers of trials per session or in total. This reasoning is in line with our finding that the magnitude of threshold decrease (especially in the late part of learning) correlates best with position and orientation specificity, and not with the number of training trials performed.

In conclusion, many factors determine the extent to which a skill remains specific after training or can generalize to other conditions. The present study suggests that the set of factors that determines the extent of generalization is at least partially different in early and late phases of visual skill learning. More research is required to identify the mechanisms contributing to early and late-phase generalization.
